# A novel partitivirus confers dual contradictory effects to its host fungus: growth attenuation and virulence enhancement

**DOI:** 10.1128/jvi.01219-25

**Published:** 2025-11-24

**Authors:** Zhen Liu, Maoqiu Chen, Ioly Kotta-Loizou, Robert H. A. Coutts, Linghong Kong, Hamdy Aboushedida, Risky Kartika Sari, Hiromitsu Moriyama, Wenxing Xu

**Affiliations:** 1 National Key Laboratory for Germplasm Innovation & Utilization of Horticultural Crops, Huazhong Agricultural University 47895, Wuhan, China; 2 Hubei Hongshan Laboratory, Huazhong Agricultural University 47895, Wuhan, China; 3 Key Laboratory of Plant Pathology of Hubei Province, Huazhong Agricultural University 47895, Wuhan, China; 4 College of Plant Science and Technology, Huazhong Agricultural University 47895, Wuhan, China; 5 Department of Life Sciences, Faculty of Natural Sciences, Imperial College London 4615, London, United Kingdom; 6 Department of Clinical, Pharmaceutical and Biological Science, School of Life and Medical Sciences, University of Hertfordshire 3769, Hatfield, United Kingdom; 7 Department of Applied Biological Sciences, Tokyo University of Agriculture and Technology 13125, Fuchu, Tokyo, Japan; Cornell University Baker Institute for Animal Health, Ithaca, New York, USA

**Keywords:** mycovirus, *Camellia sinensis*, two-sided effects, *Sinodiscula camellicola* partitivirus 1, hypervirulence, *Partitiviridae*

## Abstract

Mycoviruses possess a potential role for biological control due to their ability to reduce both virulence and vegetative growth in some phytopathogenic fungi. However, mycoviruses that enhance fungal pathogenicity have been poorly studied and characterized. In this study, a novel double-stranded RNA (dsRNA) fungal virus, tentatively named Sinodiscula camellicola partitivirus 1 (ScPV1), was identified in the phytopathogenic fungus *Sinodiscula camellicola*, isolated from tea leaves. ScPV1 possesses two genomic components of 1,835 bp and 1,697 bp in length, each containing an open reading frame (ORF) encoding a putative RNA-dependent RNA polymerase (RdRP) and coat protein (CP), respectively, as confirmed by mass spectrometry. Phylogenetic analysis of the amino acid sequences of the RdRPs from ScPV1 and related mycoviruses placed ScPV1 within a newly proposed genus, Epsilonpartitivirus, in the family *Partitiviridae*. The virus was purified using ultracentrifugation, and transmission electron microscopy revealed that ScPV1 dsRNA genomes are encapsidated in virus particles *ca*. 31 nm in size, ranging from 24.9 to 36.8 nm, together with the RdRP protein, which was of an unexpected size. Transfection with purified virions generated transfectants with significantly reduced growth rates but with increased virulence, indicating that ScPV1 confers unusual effects on its host fungus. This finding represents a significant advancement in understanding the complex interactions between mycoviruses and their host fungi.

**IMPORTANCE:**

Here, we identified a novel partitivirus, tentatively named Sinodiscula camellicola partitivirus 1 (ScPV1), marking the first report of a partitivirus from a phytopathogenic fungus infecting tea plants. ScPV1 is characterized by possession of two dsRNA genomic components encapsidated in particles of varying sizes, along with an RNA-dependent RNA polymerase protein of an expected size, which contained some unique amino acids, indicating its distinct molecular and morphological traits. Biological tests on transfectants generated following protoplast infection with purified virions demonstrated that ScPV1 impairs vegetative growth while enhancing virulence in its fungal host. This finding represents the first instance of a mycovirus responsible for hypervirulence on a phytopathogenic fungus through virion transfection, as well as the first case of a partitivirus conferring hypervirulence while reducing vegetative growth in a phytopathogenic fungus. We anticipate that these findings will significantly advance our understanding of the complex interactions between mycoviruses and their host fungi.

## INTRODUCTION

Mycoviruses (or fungal viruses) either affect the biological traits of their host fungi or are latent (1). Some viruses can impair the growth of and reduce the virulence in their host fungi, making them potential biocontrol agents. For example, Cryphonectria hypovirus 1 (CHV1) has been used to control chestnut blight in Europe, and Sclerotinia sclerotiorum hypovirulence-associated DNA virus 1 (SsHADV-1) has been applied to manage oilseed rape *Sclerotinia* stem rot in China (2, 3). Conversely, certain mycoviruses can enhance specific biological traits in their host fungi, granting them an adaptive advantage in particular environments. This is illustrated by the cases of Beauveria bassiana victorivirus 1 (BbVV-1) and Beauveria bassiana polymycovirus 1 (BbPmV-1), which, when infecting the entomopathogenic fungus *Beauveria bassiana*, lead to an increase in virulence against insects (4). This hypervirulent trait is attractive since it enhances the potential biocontrol effect for host fungi against agricultural pests. Conversely, two mycoviruses have been demonstrated to be related to hypervirulence in phytopathogenic fungi: a 6.0-kbp dsRNA, which is phylogenetically related to plant cryptic viruses, in *Nectria radicicola*, the causal fungus of ginseng root rot (5); and Leptosphaeria biglobosa quadrivirus-1 (LbQV-1) in *Leptosphaeria biglobosa*, causing *Phoma* stem canker (blackleg) of oilseed rape (6). The mycoviruses associated with hypervirulence in phytopathogenic fungi have implications that run counter to their potential use as biocontrol agents. Consequently, careful management and utilization of these mycoviruses is essential to prevent them from inadvertently increasing the virulence of their fungal hosts.

The family *Partitiviridae* contains a large number of viruses that infect fungi, plants, and protozoa and have been divided into the five genera *Alphapartitivirus*, *Betapartivirus*, *Deltapartivirus*, *Gammapartitivirus*, and *Cryspovirus* (7, 8) and the two newly proposed genera Epsilonpartitivirus (9) and Zetapartitivirus (10, 11). Members of this family possess genomes comprising two linear double-stranded RNA (dsRNA) molecules, sized between 1.4 and 2.4 kilobases (kbp), with the larger molecule encoding the RNA-dependent RNA polymerase (RdRP) and the smaller one encoding the capsid protein (CP), both of which are individually packaged into viral particles measuring 25–40 nanometers in size (8). Partitiviruses are generally known for their persistent and asymptomatic infections in their hosts (7); however, there are notable exceptions. For example, Colletotrichum alienum partitivirus 1 (CaPV1) has been shown to significantly reduce host virulence, mycelial growth, appressorial development, and appressorium turgor, while increasing conidial production with abnormal morphology (12). Similarly, Aspergillus fumigatus partitivirus 1 (13) and Heterobasidion partitivirus 3 (14) have been found to attenuate the growth rates of their host fungi, and Aspergillus flavus partitivirus 1 (10) and Sclerotinia sclerotiorum partitivirus 1 (15) have been observed to attenuate virulence. Additionally, Rosellinia necatrix partitivirus 2 (RnPV2) caused no apparent morphological changes to its host fungus *Rosellinia necatrix*, but induced obvious morphological changes in a Dicer-like 2 knockout mutant (dcl-2) of a non-natural host, *Cryphonectria parasitica,* which causes chestnut blight (16). Moreover, infection with a partitivirus has been shown to induce hypervirulence in *Talaromyces marneffei*, a thermal dimorphic clinical fungus (17). However, the extent to which partitivirus-mediated hypervirulence occurs in phytopathogenic fungi in nature is not known.

Tea, represented by the species *Camellia sinensis* (L.) Kuntze, is of considerable economic significance in China and provides a multitude of health benefits to humans. Within the spectrum of diseases that afflict tea plants, anthracnose emerges as one of the most severe, presenting a substantial challenge to the cultivation of tea (18). The pathogens responsible for tea anthracnose exhibit regional diversity across the globe, with the disease commonly attributed to *Discula theae-sinensis* (formerly known as *Gloeosporium theae-sinensis*) and various species of *Colletotrichum* (19). Recently, two new fungal species, *Sinodiscula theae-sinensis* and *Sinodiscula camellicola*, which belong to a newly proposed genus *Sinodiscula* within the family *Melanconiellaceae*, have been identified and characterized as causative agents of tea anthracnose (20). The disease caused by these fungi exhibits similar symptoms: initially, dark green or yellowish-brown watery spots appear, which then expand along the leaf veins, forming irregular-shaped spots. These spots gradually turn brown or reddish-brown and eventually become grayish-white (20). The visual similarity of the disease symptoms caused by these pathogens presents a challenge in differentiating them through unaided visual inspection. Consequently, a specific disease name, “tea leaf blight,” has been proposed to describe the symptoms induced by *Sinodiscula* species (20). Given that the majority of Chinese tea cultivars are susceptible to this fungal disease, the implementation of effective control measures is essential to protect the yield and quality of tea crops. This imperative necessitates the investigation of mycoviruses that infect related fungi as an understanding of these viral interactions could potentially lead to novel strategies for disease management.

In this study, a novel partitivirus was identified from *S. camellicola* isolated from tea plants, and its molecular, morphological, and biological traits were characterized. This partitivirus exhibits some unique molecular and morphological traits unreported in other partitiviruses and dual effects on the fungal host by simultaneously attenuating growth while enhancing virulence, marking the first instance of a partitivirus contributing to hypervirulence in a phytopathogenic fungus.

## RESULTS

### Partitivirus dsRNAs in *S. camellicola* strain WJT-1-1

**Fig 1:**
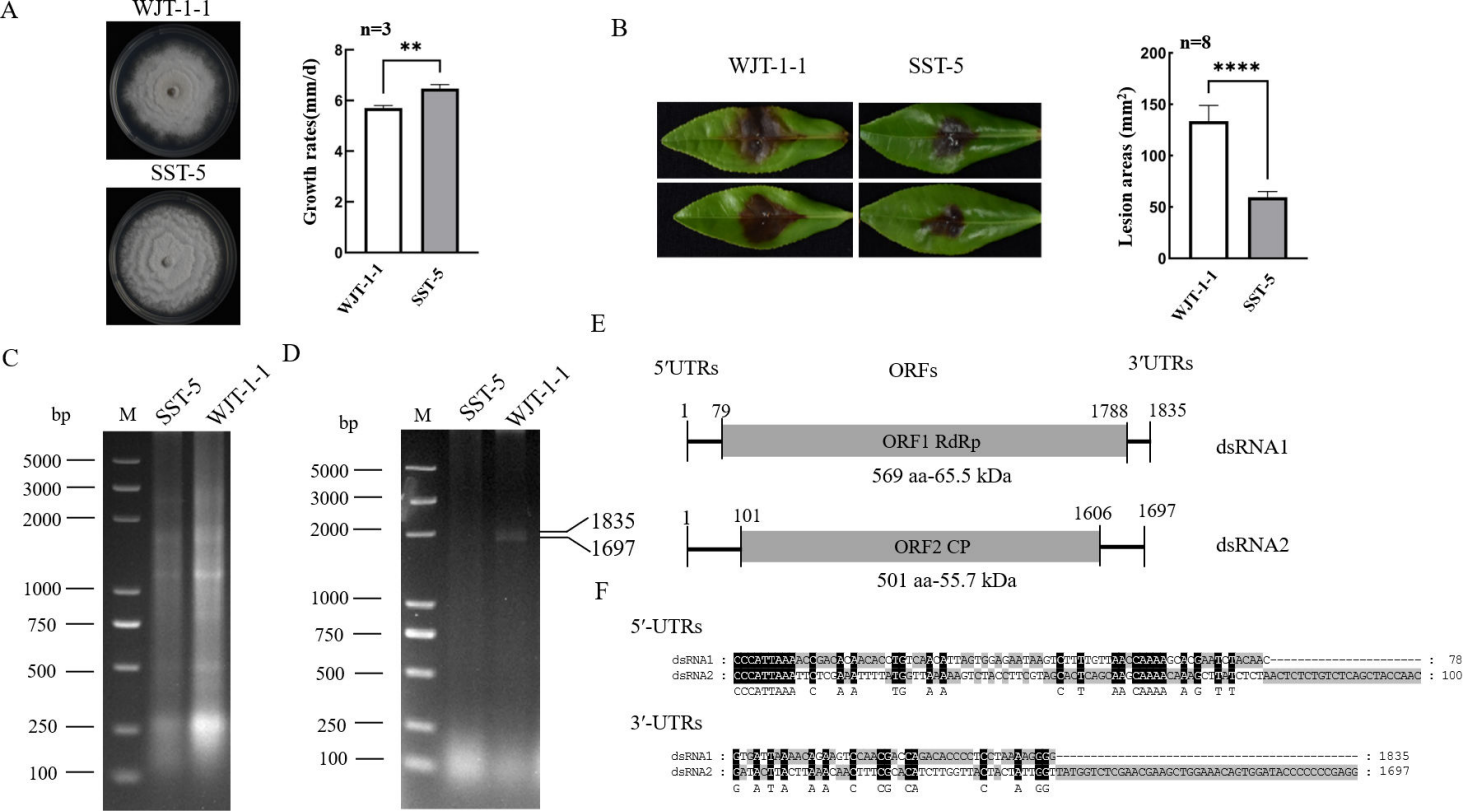
Biological tests of *S. camellicola* strains, and electrophoretic analysis and genomic organization of Sinodiscula camellicola partitivirus1 (ScPV1). (**A**) Colonies and growth rates of strains WJT-1-1 and SST-5 cultured on the PDA medium for 10 days. (**B**) The symptoms and lesion lengths caused by strains WJT-1-1 and SST-5 on tea leaves (*Camellia sinensis* cv. “Echa 1”). The significance of differences was determined by t test (**P < 0.01; ****P < 0.0001). (**C and D**) Electrophoretic profile on agarose gels of dsRNA preparations extracted from *Sinodiscula camellicola* strains SST-5 and WJT-1-1 before (**C**) and after treatment with DNase I and S1 nuclease (**D**), respectively. (**E**) Schematic representation of the genomic organization of ScPV1 RNA 1 and RNA 2. Gray boxes and black lines indicate open reading frames (ORFs) and untranslated regions (UTRs), respectively. (**F**) Sequence alignment of the UTRs of both ScPV1 dsRNAs. Black and gray backgrounds indicate conserved and semi-conserved nucleotide residues, respectively.

*S. camellicola* strain WJT-1-1 isolated from tea leaves exhibited enhanced virulence but lower growth rates as compared to other *S. camellicola* strains, as exemplified by strain SST-5 (Fig. 1A and B). To investigate whether these biological changes were due to the presence of a virus, dsRNAs were extracted from the mycelia of strains WJT-1-1 and SST-5 and subjected to electrophoresis on a 1.0% agarose gel. No significant differences were observed in their RNA patterns (Fig. 1C). Further analysis involved subjecting the nucleic acids to agarose gel electrophoresis after digestion with DNase I and S1 nuclease, revealing two dsRNA bands termed dsRNA1 and 2 based on their sizes exclusively in strain WJT-1-1 (Fig. 1D). Random primers were employed for reverse transcription and polymerase chain reaction (RT-PCR) amplification and rapid amplification of cDNA ends (RACE). The sequences of full-length complementary DNA (cDNA) clones were determined for dsRNA1 (GenBank no. PQ201539), which was 1,835 bp in length, and dsRNA2 (PQ201540), which was 1,697 bp in length (Fig. 1E). The 5′ and 3′-untranslated regions (UTRs) were 78 bp and 47 bp in dsRNA1 and 100 bp and 91 bp in dsRNA2, respectively. The 5′-UTRs exhibited conservation, featuring the conserved stretch “CCCAUUAAA.” In contrast, the 3′-UTRs of dsRNA1 and dsRNA2 showed lower conservation (Fig. 1F). Genomic organization analysis of both dsRNAs revealed that dsRNA1 contains one open reading frame (ORF) on the positive strand. This ORF starts at nucleotide (nt) 79 and terminates at nt 1,788, encoding a putative protein of 569 amino acids (aa) with an estimated molecular weight of 65.5 kDa. Similarly, ORF2 in dsRNA2 spans from nt 101 and terminates at nt 1606 and putatively encodes a protein of 501 aa with a molecular weight of 55.7 kDa.

Blastx searches revealed that the proteins encoded by dsRNA1 and dsRNA2 share the highest identity with the RdRP gene (NCBI GenBank No. UOK20169, 92% coverage, 62.70% identity, E value  =  0.0) and CP (88% coverage, 52.27% identity, E value  =  5e-160) of Diplodia seriata partitivirus 1 (DsPV1) and Colletotrichum liriopes partitivirus 1 (ClPV1) (Table S1), supporting the notion that dsRNAs 1 and 2 harbor putative ORFs encoding RdRP and CP, respectively. Additionally, the putative RdRP shares serially high identities (92%–93% coverage, 61.71%–62.46% identity, E value  =  0.0) with the RdRP of Colletotrichum eremochloae partitivirus 1, Erysiphe necator-associated partiti-like virus 1, and Metarhizium brunneum partitivirus 1 in the family *Partitiviridae* (Table S1). Based on their molecular characteristics and similarity to other viruses, these dsRNAs are proposed as a novel partitivirus, tentatively named Sinodiscula camellicola partitivirus 1 (ScPV1).

An additional 31 *S*. *camellicola* samples were collected from two distinct counties in Hubei Province: 23 from Xuan'en County (Enshi Tujia and Miao Autonomous Prefecture) and nine from Zigui County (Yichang City), in China, and subjected to ScPV1 identification by RT-PCR using the extracted nucleic acids and the primer pair nominated RNA1-F2/RNA1-R2, resulting in amplicons in size of 411 bp (Fig. S1 and Table S2). It revealed an incidence rate of 39.1% in Xuan'en County samples, while none were detected in Zigui County samples, supporting its regional prevalence in specific areas.

### Multiple alignment and phylogenetic analysis of the RdRP reveals that ScPV1 belongs to a new viral genus

**Fig 2:**
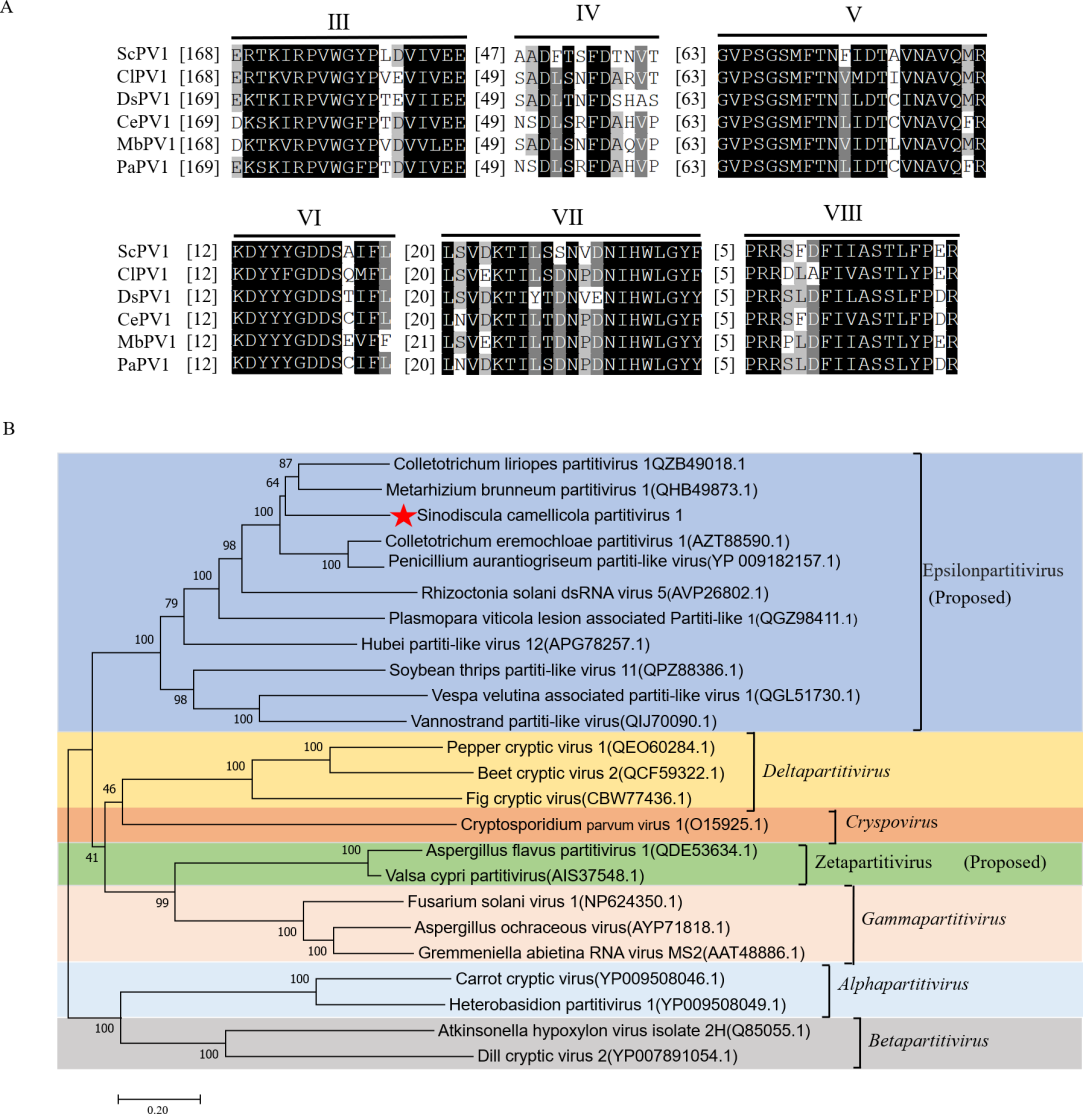
Sequence and phylogenetic analysis of the ScPV1 genome. (**A**) Multiple alignment of six conserved motifs (III to VIII) in the RdRP sequences of ScPV1 and some closely related members belonging to the family *Partitiviridae*. Black and gray backgrounds indicated conserved and semi-conserved amino acid residues, respectively. The abbreviated names refer to Colletotrichum liriopes partitivirus 1 (ClPV1, QZB49018.1); Colletotrichum eremochloae partitivirus 1 (CePV1, AZT88590.1); Metarhizium brunneum partitivirus 1 (MbPV1, QHB49873.1); Diplodia seriata partitivirus 1 (DsPV1, UOK20169.1); and Penicillium aurantiogriseum partitivirus 1 (PaPV1, AZT88600.1). (**B**) A maximum likelihood phylogenetic tree constructed using the RdRP sequences of ScPV1 and some representative members of all the genera belonging to the family *Partitiviridae*. The red pentagram indicates the position of ScPV1.

Multiple alignment analysis was performed for the putative RdRP sequences of ScPV1 and some closely related members in the family *Partitiviridae*. Six conserved motifs (Motifs III to VIII), similar to those observed in other related members, were detected, whereas ScPV1 RdRP contains several unique amino acids, including a phenylalanine (F) instead of leucine (L) in Motif IV, F instead of valine (V) or isoleucine (I) or L in Motif V, and serine (S) instead of aspartic acid (D) in Motif VII (Fig. 2A). A phylogenetic tree was constructed using the sequence of the ScPV1 RdRP gene and representative members from various genera within the family *Partitiviridae*. The analysis revealed that the ScPV1 RdRP gene clusters with those of members belonging to the newly proposed genus Epsilonpartitivirus, while also forming an independent branch within the tree (Fig. 2B). Based on the established threshold criteria for species distinction, which requires a 90% identity in RdRP sequences (21), ScPV1 is confirmed as a novel member of the genus Epsilonpartitivirus within the family *Partitiviridae*.

### ScPV1 is encapsidated in isometric particles

**Fig 3:**
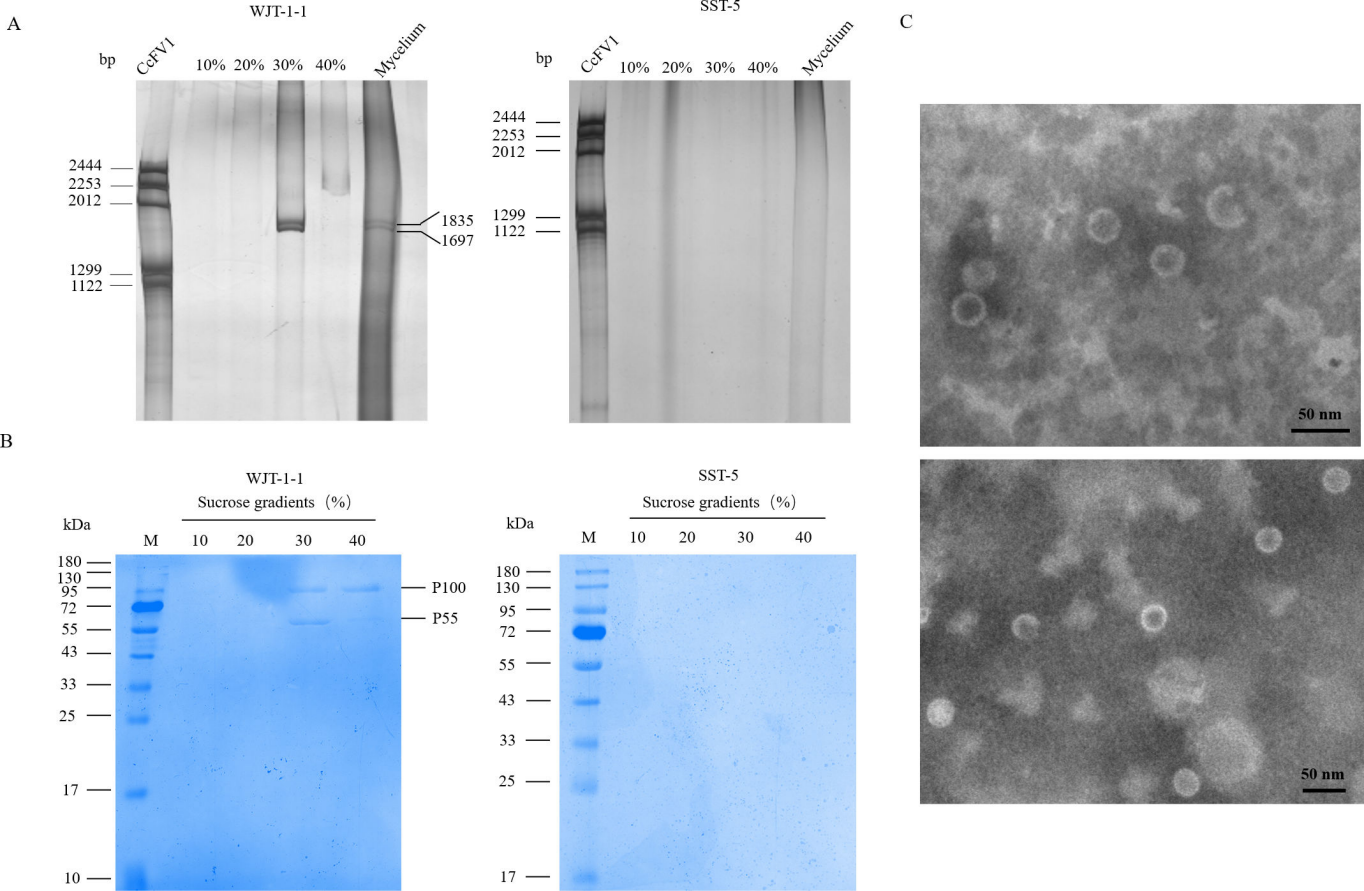
Analysis of nucleic acids and proteins associated with ScPV1 virus-like particles (VLPs). (**A**) Agarose gel electrophoresis of dsRNAs extracted from purified ScPV1 VLPs obtained from sucrose gradient fractions ranging from 100 to 400 mg/mL with increments of 100 mg/mL. Comparison of purified virus isolated from strain WJT-1-1 mycelia with virus-free strain SST-5 mycelia. Colletotrichum camelliae filamentous virus 1 (CcFV-1) genomic components were used as dsRNA markers. (**B**) SDS-PAGE of proteins extracted from the aforementioned sucrose gradient fractions. M, protein molecular weight marker. (**C**) Representative TEM images of VLPs purified from strain WJT-1-1.

The encapsidation of ScPV1 dsRNA in particles was investigated using virions purified from strains WJT-1-1 and SST-5, which were subjected to stepwise sucrose gradient centrifugation (10% to 40% in 10% sucrose increments). Nucleic acids were extracted from each gradient fraction and subjected to electrophoretic analysis on 6% polyacrylamide gel electrophoresis (PAGE) gels, the results of which showed that the target dsRNA bands, which matched the sizes of those extracted from strain WJT-1-1 mycelia exclusively detected in nucleic acid preparations of strain WJT-1-1, sharply concentrated in the 30% sucrose fraction (Fig. 3A).

To identify presumed virion proteins, these were extracted from 10% to 40% sucrose gradient fractions and individually subjected to SDS-PAGE. The results indicated that two protein bands, designated as P55 and P100 according to their estimated sizes, were predominantly found in the 30% sucrose fraction. Additionally, a protein band matching the size of P100 was specifically concentrated in the 40% sucrose fraction. Both protein bands were excised and analyzed using peptide mass fingerprinting (PMF). The results revealed that 228 peptide segments from P55 matched the protein sequence encoded by dsRNA2, representing approximately 74% of the entire sequence (Table S3), confirming that P55 is the CP encoded by dsRNA2. Additionally, seven peptide segments from P100 matched the protein sequence encoded by dsRNA1, accounting for about 20% of the entire sequence (Table S4), indicating that P100 is likely the RdRP protein encoded by dsRNA1. No proteins were detected in the ScPV1-free strain SST-5 (Fig. 3B). Transmission electron microscopy (TEM) examination of these fractions revealed the presence of isometric VLPs, having a diameter that spanned from 24.9 to 36.8 nm, with an average of 31 nm (Fig. 3C). In contrast, no viral particles were discerned in the control strain SST-5.

### ScPV1 infection reduces growth rate but increases host fungus virulence

To assess viral transmission, the ScPV1-infected strain WJT-1-1, acting as the donor of the virus, was cultured in direct contact with the uninfected strain SST-5, which served as the recipient. Following a 7-day contact-culture period, 24 mycelial disks (marked by asterisks in Fig. S2A) were excised from the colonies of strain SST-5 at six independent peripheral positions on the colonies and subjected to dsRNA extraction. After treatment with the S1 enzyme, the extracted dsRNAs were visualized on agarose gels, and the results showed the absence of any dsRNAs in the strain SST-5 sub-isolates, suggesting that ScPV1 is most likely difficult to be horizontally transmitted from strain WJT-1-1 to other strains of the fungus (Fig. S2B).

**Fig 4:**
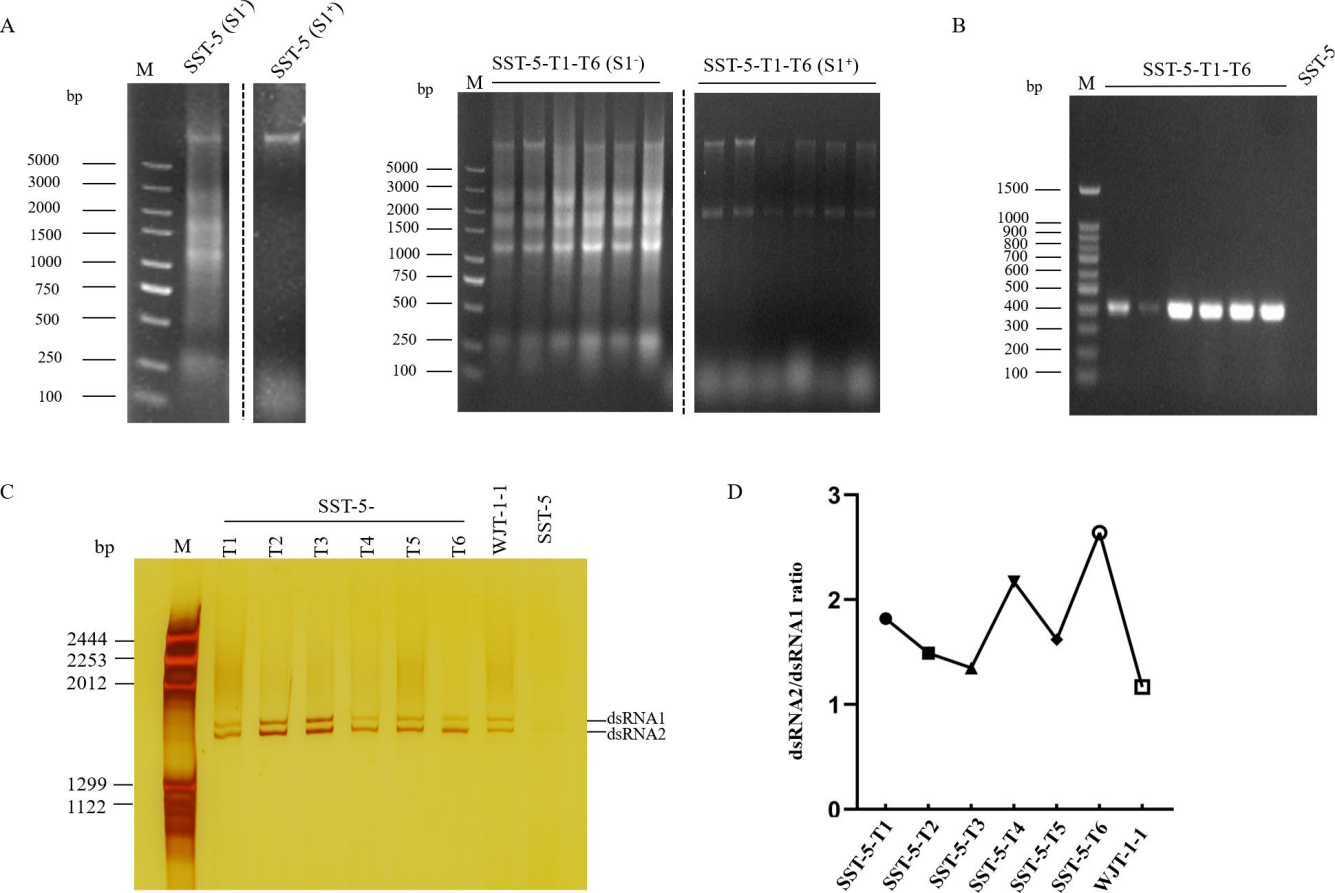
Horizontal transmission of ScPV1 via co-cultivation with an uninfected strain and the dsRNA2-to-dsRNA1 ratios of ScPV1 before and after transfection. (**A**) Nucleic acid extracts from strain SST-5 and its ScPV1 transfectants (SST-5-T1 to -T6), either untreated (S1^−^) or treated with S1 nuclease (S1^+^). The high-molecular-weight bands (> 5,000 bp) correspond to fungal genomic DNA. (**B**) RT-PCR confirmation of ScPV1 infection in transfectants (SST-5-T1 to -T6). The parental strain SST-5 served as a negative control. (**C**) Non-denaturing PAGE of dsRNA preparations extracted from *S. camellicola* strains SST-5, WJT-1-1, and transfectants SST-5-T1 to -T6 after treatment with DNase I and S1 nuclease, respectively. Colletotrichum camelliae filamentous virus 1 (CcFV-1) genomic components were used as dsRNA markers. (**D**) The dsRNA2/dsRNA1 ratios in WJT-1-1 and the transfectants, as shown in the PAGE gel.

To further assess the biological consequences of ScPV1 infection, purified virus particles were transfected into the protoplasts of strain SST-5 using PEG mediation. A total of 110 protoplast-generated colonies were randomly picked and transferred on fresh plates incubated as above and subjected to nucleic acid extraction. The extracted nucleic acids were treated with S1 enzyme and analyzed by agarose gel electrophoresis, which revealed that six transfectants (representing a frequency of 5%) were infected with ScPV1 (Fig. 4A). This finding was further supported by RT-PCR identification using the extracted nucleic acids and the primer pair RNA1-F2/RNA1-R2 (Fig. 4B; Table S2).

To determine the ratios of dsRNA1 to dsRNA2 in the transfectants, strains SST-5-T1 to SST-5-T6 together with WJT-1-1 were subjected to dsRNA extraction, followed by DNase I and S1 nuclease treatment, and non-denaturing PAGE analysis. The results revealed that the dsRNA2/dsRNA1 ratios varied among transfectants, ranging from 1.32 in SST-5-T3 to 2.68 in SST-5-T6, but were consistently higher than the ratio (1.19) observed in the original strain WJT-1-1 (Fig. 4C and D).

**Fig 5:**
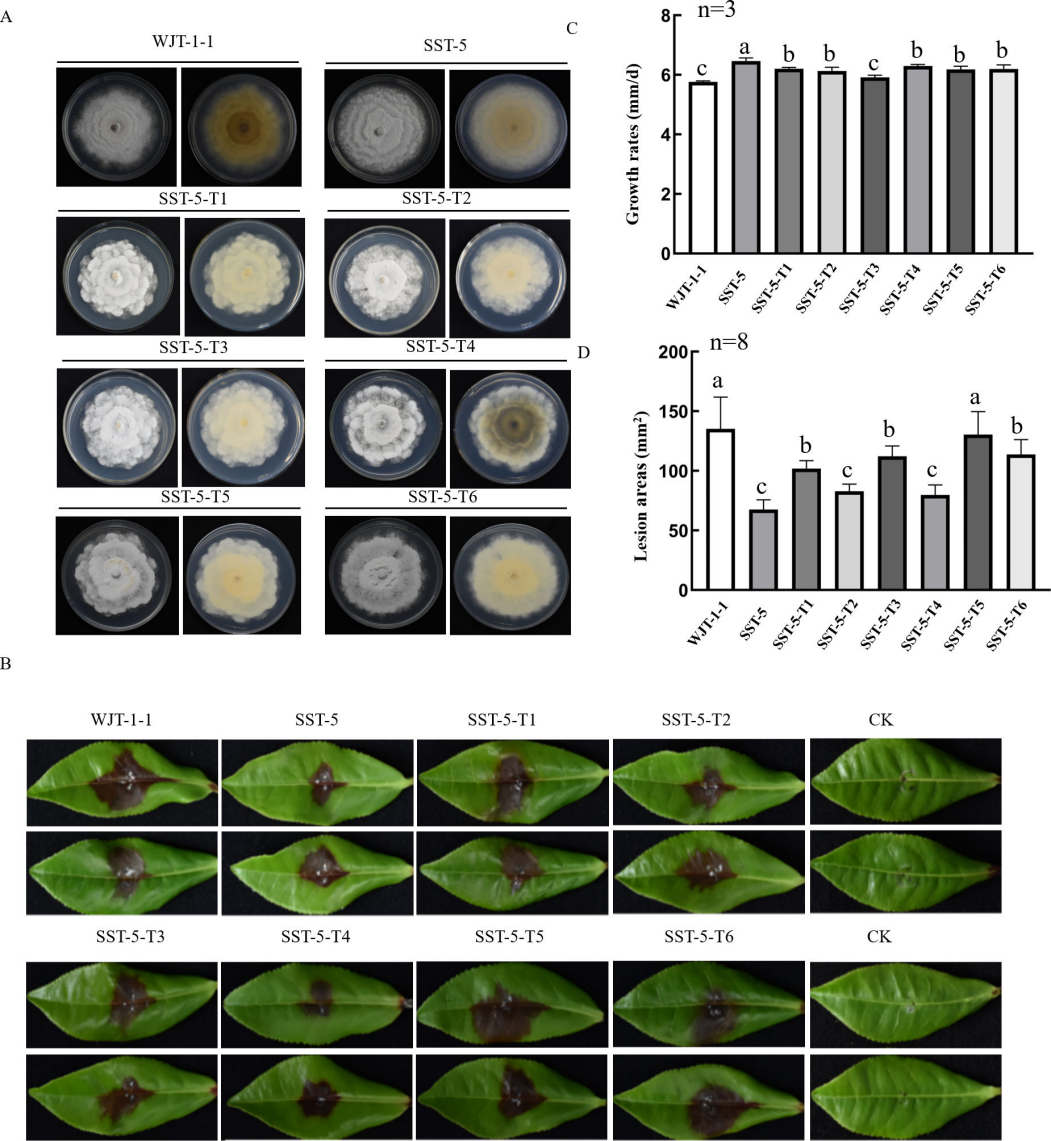
Assessment of the effects of ScPV1 on fungal morphology, growth, and pathogenicity by virus transfection. (**A**) Colony morphologies of strain SST-5 and transfectants (SST-5-T1 to -T6) infected with ScPV1, together with strain WJT-1-1 grown on PDA at 25°C in darkness for 10  days. (**B**) Representative symptoms on tea leaves (*C. sinensis* cv. “Echa 1”) following inoculation with the aforementioned ScPV1-infected transfectants, strain WJT-1-1, and ScPV1-free ScPV1 at 7 dpi. (**C and D**) Growth rates (**C**) and the resulting lesion areas (**D**) of the aforementioned strains; columns indicate average values of independent repeats (N), the different letters refer to significant difference, and error bars represent standard deviation. Data are means ± standard error of mean (SEM). Different letters indicate significant difference at *P* < 0.05 (one-way ANOVA).

Following culture on PDA, all the transfectants exhibited sparse hyphal patterns with petal-shaped edges, similar to the morphologies of strain WJT-1-1 (Fig. 5A). Furthermore, all the transfectants demonstrated reduced growth rates, ranging from 5.9 to 6.3 mm/day, as compared to their parent strain SST-5, which exhibited a growth rate of 6.5 mm/day (Fig. 5C). By contrast, most of the transfectants exhibited significantly increased virulence, resulting in lesions with areas of 102.0 to 130.4 mm^2^ except for two transfectants (SST-5-T2 and -T4), which caused similar lesions (79.7 to 83.0 mm^2^), as compared to the lesions (67.7 mm^2^) elicited by strain SST-5 at 5 days post inoculation (dpi) on *C. sinensis* cultivar “Echa” tea leaves (Fig. 5B and D).

**Fig 6:**
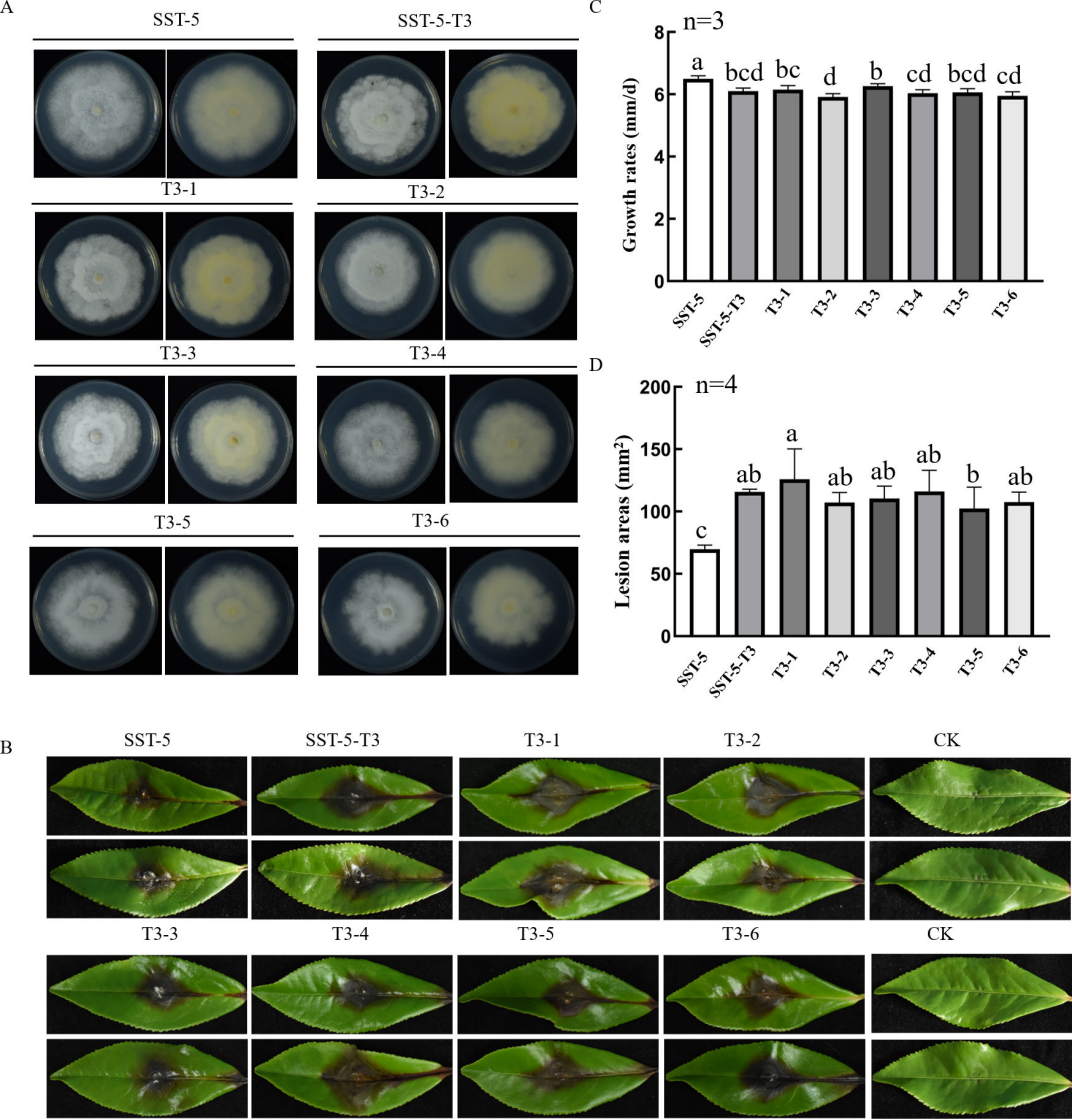
Biological effects of ScPV1 reintroduction into fungal strain SST-5 from transfectant SST-5-T3. (**A**) Colony morphologies of SST-5, its subisolates (T3-1 to T3-6) generated through contact culture with SST-5-T3, and SST-5-T3 on PDA after 10 days at 25°C in darkness. (**B**) Disease symptoms on tea leaves (*C. sinensis* cv. “Echa 1”) at 7 days post-inoculation (dpi). (**C, D**) Quantitative analysis of growth rates (**C**) and lesion areas (**D**). Bars represent means ± SEM of three biological replicates (growth rate) or four replicates (virulence). Different lowercase letters indicate statistically significant differences (*P* < 0.05, one-way ANOVA with Tukey’s *post hoc* test).

The observed biological effects might be influenced by intra-isolate variability, so the transfectant SST-5-T3, displaying relatively pronounced phenotypic alterations, was selected as the donor strain for back-introduction of ScPV1 into the virus-free recipient strain SST-5 through contact culture in four replicates (Fig. S3A). After three rounds of subculturing, RT-PCR analysis of twelve SST-5 subisolates confirmed ScPV1 infection in all cases (Fig. S3B). Six transfectants derived from SST-5-T3 (T3-1 to T3-6) were subjected to biological characterization. These transfectants exhibited reduced growth rates ranging from 5.9 to 6.3 mm/day compared to the parent strain SST-5 (6.5 mm/day; Fig. 6A and C). Furthermore, they demonstrated significantly enhanced virulence, causing lesions measuring 102.5 to 130.0 mm² on *C. sinensis* “Echa” tea leaves, whereas the parent strain SST-5 produced lesions averaging only 69.7 mm² (Fig. 6B and D).

## DISCUSSION

In this study, a new mycovirus, tentatively named ScPV1, was identified and characterized from *S. camellicola* isolated from tea plants. According to the species demarcation criteria within the family *Partitiviridae* (less than 90% aa sequence identity in the RdRP (21), as well as its genomic organization and phylogenetic analysis, ScPV1 is proposed as a new member belonging to the newly proposed genus Epsilonpartitivirus in the family *Partitiviridae*. To date, five mycoviruses, namely, Colletotrichum camelliae filamentous virus 1 (CcFV1, belonging to the family *Polymycoviridae*), Pestalotiopsis theae chrysovirus 1 (PtCV1, *Chrysoviridae*), Pestalotiopsis fici hypovirus 1 (PfHV1, *Hypoviridae*), Melanconiella theae mitovirus 1 (MtMV1, *Mitoviridae*), and Didymella theifolia botybirnavirus 1 (DtBRV1, *Botybirnaviridae*) have been identified from phytopathogenic fungi infecting tea plants, including *Colletotrichum camelliae*, *Pestalotiopsis theae*, *Pestalotiopsis fic*i, *S. camellicola,* and *Didymella theifolia*, respectively, while no partitiviruses have been reported in fungi infecting tea plants (22–26). To our knowledge, this is the first report of a partitivirus from a phytopathogenic fungus infecting tea plants.

The genomic sequences of two dsRNA fragments in ScPV1, nominated dsRNAs1 and 2, were determined with sizes of 1,835 bp and 1,697 bp, respectively. These genomes conform to the genomic size range of the family *Partitiviridae,* whose members typically have 1 or 2 segments of 1.4–2.3 kbp per segment (27). Each ScPV1 dsRNA segment contains a single ORF, encoding RdRP and CP in dsRNA1 and dsRNA2, respectively, a common feature within the *Partitiviridae* family (28). Following purification of ScPV1 from strain WJT-1-1 and application of the same procedure for strain SST-5, icosahedral virus-like particles *ca*. 31 nm in diameter were visualized *via* TEM in the mycelia of strain WJT-1-1 exclusively (Fig. 3C). Additionally, dsRNAs 1 and 2 were concentrated in the 30% fraction of sucrose gradients where both virus-like particles and structural proteins were detected (Fig. 3C; Tables S3 and S4). Additionally, P55, which corresponds in size to the CP encoded by ScPV1 dsRNA2, was identified through SDS-PAGE and PMF analysis (Fig. 3B; Table S3). These confirm that ScVP1 dsRNAs 1 and 2 are encapsidated in 31 nm viral particles encapsidated by P55. The major structural protein encoded by ScPV1 dsRNA 2 is an icosahedral T = 1 CP consisting of 60 polypeptide subunits. These observations match previous results of investigations on partitivirus particle structure including Fusarium poae virus 1 and Penicillium stoloniferum viruses S and F, characterized by cryo-electron microscopy (cryo-EM), three-dimensional image reconstruction, and X-ray crystallography (29–31). TEM observation demonstrated that ScPV1 consists of isometric particles 24.9–36.8 nm in diameter, fitting the size range (25–40 nm) for known members of the *Partitiviridae* that are variable (12). ScPV1 particles also contain a P100 protein that was detected following SDS-PAGE and PMF analysis. These studies revealed that P100 generated six peptide fragments that matched the deduced ScPV1 RdRP sequence at amino acid positions 64–217, accounting for 20% of the entire coverage. These results suggest that P100 is most likely the viral RdRP, which was significantly larger than the predicted molecular weight ScPV1 RdRP concentrated in sucrose gradients in the 30% fraction with a molecular weight of 65.5 kDa, possibly due to some processing modifications. Notably, it was observed at a significantly higher level in the 40% fraction as compared with the concentration of CP bands on SDS-PAGE gels (Fig. 3B). Typically, one or two RdRP molecules are packaged in each partitivirus particle (8), so the peptide concentration ratio should be *ca*. 60:1 or 30:1, respectively, for CP versus RdRp in partitiviruses. Multiple alignment of the ScPV1 RdRP sequence with the RdRP sequences of related dsRNA viruses indicates six conserved motifs, namely, motifs III to VIII, and the triplet "GDD" in Motif-VI, which is conserved in the RdRP sequences of +ssRNA and most dsRNA viruses (Fig. 2A) (32); however, some unique amino acids within Motifs IV, V, and VII were observed in the ScPV1 RdRP.

Fungal partitiviruses are transmitted intracellularly during cell division, hyphal anastomosis, and sporogenesis, and alpha- and betapartitiviruses infecting fungal hosts from the genus *Heterobasidion* are transmitted *via* hyphal contacts between somatically incompatible host strains (7). Here, horizontal transmission was assessed using two different strains, WJT-1-1 and SST-5, but was unsuccessful. However, it occurred at a high frequency (over 90%) between exogenous strains SST-5 and its transfectant SST-5-T3. This suggests that horizontal transmission of ScPV1 is strain-dependent. To date, virus transfection of protoplasts with partitiviruses has been reported only in a few fungal species, including *R. necatrix*, *Aspergillus fumigatus*, and *S. sclerotiorum* (13, 15, 16, 33). In this study, virus transfection was achieved using purified particles, and ScPV1 was shown to be transmitted at a low frequency of 5%, which facilitates a critical assessment of the biological traits of ScPV1. Analysis of the transfectants revealed significant variation in the relative abundance ratios of the two ScPV1 genomic segments among different clones. However, all transfectants maintained consistently higher segment ratios compared to the original WJT-1-1 strain, where the two segments showed nearly equivalent band intensities. Similarly, some transfectable partitviruses changed their dsRNA1-to-dsRNA2 ratios before and after transfection (16, 34). Multipartite (+)ssRNA viruses typically maintain a defined ratio of encapsidated genomic segments in their hosts (35), a pattern also observed in many partitiviruses (14, 36). The genomic segment ratios may affect the biological properties of ScPV1.

Most of the viruses in the family *Partitiviridae* are latent in their host fungi, but some can attenuate fungal virulence and have potential as biological control agents. For example, ClPV1 reduces virulence and sporulation of *Colletotrichum liriopes* (37); CaPV1 significantly decreases host virulence, mycelial growth, appressorial development, and appressorium turgor but increases conidial production with an abnormal morphology (12). Additionally, a gammapartitivirus reduced *C. acutatum* sporulation (38). Nearly all investigations have concentrated on partitivirus-elicited hypovirulence rather than hypervirulence, which has only been reported rarely in any phytopathogenic fungi, but has been found to enhance the virulence of *T. marneffei*, which is a dimorphic clinical fungus causing systemic mycosis in Southeast Asia (17). To our knowledge, there are very limited reported cases linking mycoviruses to the hypervirulence of fungi. In one case, a 6.0 kbp dsRNA has been shown to enhance the virulence of *N. radicicola* when horizontally transmitted into fungal strains, while the fungal virulence decreased following elimination of the dsRNA (5). In the other case, LbQV-1 causes hypervirulence together with significant alterations in pigmentation and rapid growth, as demonstrated by comparisons between virus-infected and virus-cured isogenic lines of *L. biglobosa* (6). Besides, there are several hypervirul cases reported in entomopathogenic fungi, including a mycovirus infecting *B. bassiana* (39), and one gammapartitivirus named Metarhizium flavoviride partitivirus 1 in *Metarhizium flavoviride* (40). In this study, ScPV1 was transmitted to another fungal strain using protoplast transfection with purified virions. Most of the transfectants showed lower growth rates and hypervirulent traits as compared with those of the parent strain, indicating that ScPV1 confers two contradictory effects: growth attenuation and virulence enhancement in *S. camellicola*. We also observed that two transfectants (SST-5-T2/T4) caused shorter lesions as compared with other transfectants, similar to those of the parental strain, which is a likely phenomenon observed in fungi as intra-isolate variability in different SST-5 derivatives. For example, 34 subisolates of *Botrytis porri* strain Bc-72 infected by Botrytis porri RNA virus varied greatly both in mycelial growth rates (1.0 to 6.4 mm/day) on PDA and in leaf lesion lengths (0 to 3.6 mm) on garlic leaves (41). To rigorously evaluate ScPV1’s biological effects, the virus was reintroduced into the ScPV1-free SST-5 strain through anastomosis using transfectant SST-5-T3 (which exhibited the most pronounced phenotypic alterations). All resulting subisolates consistently displayed reduced growth rates coupled with enhanced virulence compared to the parental strain (Fig. 6A through D). Thus, we provide compelling evidence to demonstrate the first instance of a mycovirus, specifically a partitivirus, conferring hypervirulence to a phytopathogenic fungus through protoplast transfection with mycoviral virions. Most mycoviruses usually confer synergistic effects by attenuating the virulence and reducing the growth to their fungal host, and only Alternaria alternata chrysovirus 1 (AaCV1) was reported to cause dual effects in its host fungus (*Alternaria alternata*), which was evaluated by comparison of several virus-high-titer and virus-low-titer isolates of AaCV1-bearing *A. alternata,* in accordance with a 13-fold increase in the AK-toxin level (42, 43). Besides, it was demonstrated that Phytophthora infestans RNA virus 2 (PiRV-2) could stimulate sporangia production but reduced vegetative growth in oomycete *Phytophthora infestans* between the isogenic PiRV-2-infected and -free isolates that were generated *via* hyphal anastomosis (44). To our knowledge, this is the first instance of a mycovirus responsible for hypervirulence on a phytopathogenic fungus through virion transfection and the first case of a partitivirus conferring dual contradictory effects (hypervirulence but reduced growth) in a phytopathogenic fungus.

In summary, a novel mycovirus nominated ScPV1, which belongs to the newly proposed genus Epsilonpartitivirus within the family *Partitiviridae*, has been identified and characterized from *S. camellicola* isolated from tea plants. This marks the first report of a partitivirus from a phytopathogenic fungus infecting tea. ScPV1 contains two dsRNA segments that encode an RdRP and a CP, respectively, and these segments are encapsidated into isometric virions of different sizes. Additionally, the RdRP exhibits an unexpected size and contains some unique amino acids, indicating that the virus possesses unique molecular and morphological characteristics. Biological tests conducted with transfectants generated through protoplast transfection with purified virions rigorously assessed the effects of ScPV1 on its host and demonstrated that ScPV1 impairs *S. camellicola* growth while simultaneously enhancing its virulence. This represents the first report of a mycovirus causing hypervirulence and conferring dual effects in a phytopathogenic fungus as demonstrated through virion transfection. This study is expected to expand our understanding of viral diversity, evolution, and biological traits of the family *Partitiviridae*.

## MATERIALS AND METHODS

### Fungal strains and cultures

*S. camellicola* strains WJT-1 to WJT-27 (including WJT-1-1) were isolated from tea gardens across a village in Xuan'en County, Enshi Tujia and Miao Autonomous Prefecture, Hubei Province, China. Strains SST, SS-1 to SS-3, JYC-1-1 to JYC-1-4, and LLX were collected from various villages in Zigui County, Yichang City, Hubei Province. All strains were isolated from diseased tea leaves showing the typical leaf blight symptoms, with the exception of strain SST, which originated from an asymptomatic tea leaf. All fungal isolates were cultured on potato dextrose agar (PDA) comprising 20% [wt/vol] diced potatoes, 2% glucose, and 1.5% agar) medium at room temperature (−25°C) unless otherwise stated.

### Extraction and validation of strain dsRNA

For dsRNA extraction, mycelial plugs of the isolates were placed on sterilized cellophane disks on PDA plates and allowed to grow for 3–5 days. Frozen mycelial powder (0.5 g) was then suspended in SDS buffer. The column method was carried out to extract total RNA of the strain as previously described (45), which was eluted with 30 µL of RNase-free water. Residual DNA and ssRNA were eliminated from the extracted nucleic acids using 2U of DNase I (Simgen, Hangzhou, China) and 10 U of S1 nuclease (TaKaRa, Dalian, China) at 37°C for 1 h. Purified dsRNA (1 µL) was subjected to electrophoretic separation on 1.2% (wt/vol) agarose gels and Tris-acetate-EDTA (TAE) buffer. The dsRNA in individual fractions was stained with ethidium bromide. ScPV1 genomic dsRNAs were then excised from the gel, purified using the DNA Gel Extraction Kit (Simgen, Hangzhou, China), dissolved in RNase-free water, and stored at −70°C until use.

### Full-length genome amplification and sequencing

The sequences of the two genomic dsRNAs of ScPV1 were determined by cloning and sequencing processes involving the generation of amplicons by RT-PCR employing the random primers 05RACE-3RT and 05RACE-3 (Table S2), as previously described (46). The terminal sequences at both 5′ and 3′ dsRNA termini were determined following cloning and sequencing of RT-PCR amplicons produced via a standardized RNA ligase-mediated rapid amplification of cDNA ends (RLM-RACE) protocol, as detailed in Table S2. The oligonucleotide primers utilized in the RLM-RACE process were designed based on the sequence data obtained from randomly primed amplicons (47). The recombinant clones were sequenced by Sanger sequencing (Sangon Biotech Company, Ltd., Shanghai, China) using the universal primer pair M13F-47/M13R-48. At least three independent clones were sequenced.

### Sequence assembly and phylogenetic analysis

Sequences were analyzed and assembled with SnapGene software. The full-length sequences were searched for sequence homology comparison using the BLAST program of the NCBI database (http://www.ncbi.nlm.nih.gov). ORFfinder (https://www.ncbi.nlm.nih.gov/orffinder/) was used to predict potential ORFs. Nucleic acid sequences and amino acid sequences were multiplexed using the software MAFFT (https://www.ebi.ac.uk/jdispatcher/msa/mafft), and GeneDoc was used to visualize the results. Phylogenetic tree analysis was performed using MEGA 7 software based on the coding virus RdRP nucleotide sequence using the neighbor-joining method (48). Self-expansion support of the generated phylogenetic trees was examined with the Bootstrap program (1,000 replicates). The online websites Phyre2 (http://www.sbg.bio.ic.ac.uk/phyre2/) and SMART (http://smart.embl-heidelberg.de/) were used to predict conserved protein structural domains.

### Purification analysis and electron microscopic observation of virus particles

Extraction of ScPV1 virus particles from strain WJT-1-1 and negative control strain SST-5 was performed using ultrafast gradient centrifugation, as previously described (49). Briefly, ca. 30 g mycelia were mixed with 100 mM phosphate buffer (PB; 8.0 mM Na_2_HPO_4_, 2.0 mM NaH_2_PO_4_, pH 7.4) at a ratio of 4 mL/g of mycelia, followed by centrifugation at 10,000 rpm/min for 30 min at 4°C to eliminate cellular debris. Subsequently, the supernatant was subjected to ultracentrifugation (utilizing the Optima LE-80K system from Beckman Coulter, Inc.) at 26,000 rpm for 3 h at 4°C, enabling the precipitation of viral particles. These particles were then resuspended in a 100 mM PB. Further purification of the crude viral preparation was achieved through sucrose gradient centrifugation (50). Subsequently, aliquots of 100 µL from each fraction underwent dsRNA extraction to ascertain the presence of viral dsRNAs. The purified virion solution was adsorbed onto a copper mesh for 3 min, and then the mesh was placed on absorbent filter paper and allowed to dry for 2 min. Finally, the mesh was placed in a negative staining solution for 3 min and dried for 2 min for electron microscopic observation (51). The length of the observed viruses was measured using ImageJ software (52).

### SDS-polyacrylamide gel electrophoresis

Proteins isolated from distinct sucrose gradient fractions underwent comprehensive fractionation procedures utilizing 12% SDS-PAGE. This electrophoretic analysis was performed in a buffer system composed of 25 mM Tris/glycine and 0.1% SDS, which provided optimal conditions for protein separation. The resulting gels were subjected to staining with Coomassie brilliant blue R-250 (Bio-Safe CBB; Bio-Rad, USA), and individual protein bands were excised for PMF analysis by Sangon Biotech, Co., Ltd, in Shanghai, China (41).

### Analysis of dsRNAs on horizontal transmission

Dual cultures were used to determine the potential horizontal transfection of fungi with dsRNA viruses (53). Mycelial disks (5 mm diameter) from ScPV1-infected donor strains and virus-free recipient strains were co-cultured in paired combinations on 9 cm Petri dishes, maintained at 25°C with four or six biological replicates per treatment. After 7 days incubation, mycelial clusters were collected from the peripheral hyphal growth area of the recipient strain and sub-cultured onto PDA plates covered with sterilized cellophane. Following an additional 7 days of incubation at 25°C in a constant temperature incubator, the cultured mycelia were harvested and subjected to dsRNA extraction.

### Biological testing

Fungal morphologies and growth rates were evaluated as previously described (54). Virulence tests were conducted on fresh detached tea leaves (*C. sinensis* cv. “Echa 1”) in six replicates. Briefly, tea leaves were subjected to a three-step washing process with sterile water, followed by air-drying, prior to being inoculated. The leaves were pricked three times with a sterilized needle (0.5 mm in diameter) and inoculated with a mycelial disk. The inoculated leaves were incubated at 25°C, and disease progress was recorded every 24 h. The lesion areas were measured using ImageJ software, and the data were recorded.

### Preparation of protoplasts and transfection of virus particles

Protoplasts were prepared from the fresh mycelium of the ScPV1-free *S. camellicola* strain SST-5 that were growing vigorously as described previously (55). Briefly, *ca*. 0.5 g mycelia were mixed with 10 mL NaCl buffer (700 mM NaCl, 0.1 g lysing enzymes from *Trichoderma harzianum* and 0.01 g snailase, followed by incubation for 3 h at 30°C with shaking at 100 rpm/min to obtain protoplasts. Subsequently, protoplasts were filtered using a Millipore filter and counted with a hemocytometer slide, and 2.0 × 10^6^ protoplasts were used for each transfection. ScPV1 virions (*ca*. 70.0–80.0 µg) were transfected using PEG 6000 as previously described (56). Following transfection, protoplast suspensions were evenly spread on Bottom Agar plates (200 g of sucrose, 3 g of yeast extract per liter, 3 g casein acid hydrolysate, and 10 g agar) and incubated at 25°C.

### Statistical analysis

The biological data were statistically analyzed using SPSS Statistics Software 27.0 with one-way analysis of variance (ANOVA) and comparison of means. The mean values for the biological replicates are presented as column charts with error bars representing SEM. The graphs were generated using both MS Excel and GraphPad Prism 7 (a software program provided by GraphPad). *P*-values < 0.05 were considered to indicate statistical significance.

## Data Availability

Sequence data supporting the findings of this study have been deposited in GenBank under the accession numbers PQ201539 and PQ201540 for ScPV1. The remaining data are available within the article and supplemental material and from the corresponding author upon request.

## SUPPLEMENTAL MATERIAL

Supplemental material jvi.01219-25-s0001.docx Figures S1 to S3; Tables S1 to S4.

ASM does not own the copyrights to Supplemental Material that may be linked to, or accessed through, an article. The authors have granted ASM a non-exclusive, world-wide license to publish the Supplemental Material files. Please contact the corresponding author directly for reuse.

## References

[1] Myers JM, James TY. 2022. Mycoviruses. Curr Biol 32:R150–R155. doi:10.1016/j.cub.2022.01.049 35231405

[2] Yu X, Li B, Fu Y, Jiang D, Ghabrial SA, Li G, Peng Y, Xie J, Cheng J, Huang J, Yi X. 2010. A geminivirus-related DNA mycovirus that confers hypovirulence to a plant pathogenic fungus. Proc Natl Acad Sci USA 107:8387–8392. doi:10.1073/pnas.0913535107 20404139 PMC2889581

[3] Heiniger U, Rigling D. 1994. Biological control of chestnut blight in Europe. Annu Rev Phytopathol 32:581–599. doi:10.1146/annurev.py.32.090194.003053

[4] Filippou C, Garrido-Jurado I, Meyling NV, Quesada-Moraga E, Coutts RHA, Kotta-Loizou I. 2018. Mycoviral population dynamics in Spanish isolates of the entomopathogenic fungus Beauveria bassiana. Viruses 10:665. doi:10.3390/v10120665 30477213 PMC6315922

[5] Ahn IP, Lee YH. 2001. A viral double-stranded rna up regulates the fungal virulence of Nectria radicicola . MPMI 14:496–507. doi:10.1094/MPMI.2001.14.4.496 11310737

[6] Shah UA, Kotta-Loizou I, Fitt BDL, Coutts RHA. 2019. Identification, molecular characterization, and biology of a novel quadrivirus infecting the phytopathogenic fungus Leptosphaeria biglobosa. Viruses 11:9. doi:10.3390/v11010009 PMC6356713 30585188

[7] Vainio EJ, Chiba S, Ghabrial SA, Maiss E, Roossinck M, Sabanadzovic S, Suzuki N, Xie J, Nibert M, Ictv Report Consortium. 2018. ICTV virus taxonomy profile: Partitiviridae. J Gen Virol 99:17–18. doi:10.1099/jgv.0.000985 29214972 PMC5882087

[8] Nibert ML, Ghabrial SA, Maiss E, Lesker T, Vainio EJ, Jiang D, Suzuki N. 2014. Taxonomic reorganization of family Partitiviridae and other recent progress in partitivirus research. Virus Res 188:128–141. doi:10.1016/j.virusres.2014.04.007 24768846

[9] Nerva L, Silvestri A, Ciuffo M, Palmano S, Varese GC, Turina M. 2017. Transmission of Penicillium aurantiogriseum partiti-like virus 1 to a new fungal host (Cryphonectria parasitica) confers higher resistance to salinity and reveals adaptive genomic changes. Environ Microbiol 19:4480–4492. doi:10.1111/1462-2920.13894 28836717

[10] Jiang Y, Wang J, Yang B, Wang Q, Zhou J, Yu W. 2019. Molecular characterization of a debilitation-associated partitivirus infecting the pathogenic fungus Aspergillus flavus. Front Microbiol 10:626. doi:10.3389/fmicb.2019.00626 30984147 PMC6447663

[11] Gilbert KB, Holcomb EE, Allscheid RL, Carrington JC. 2019. Hiding in plain sight: new virus genomes discovered via a systematic analysis of fungal public transcriptomes. PLoS One 14:e0219207. doi:10.1371/journal.pone.0219207 31339899 PMC6655640

[12] Zhu JZ, Qiu ZL, Gao BD, Li XG, Zhong J. 2024. A novel partitivirus conferring hypovirulence by affecting vesicle transport in the fungus Colletotrichum. mBio 15:e0253023. doi:10.1128/mbio.02530-23 38193704 PMC10865989

[13] Bhatti MF, Jamal A, Petrou MA, Cairns TC, Bignell EM, Coutts RHA. 2011. The effects of dsRNA mycoviruses on growth and murine virulence of Aspergillus fumigatus. Fungal Genet Biol 48:1071–1075. doi:10.1016/j.fgb.2011.07.008 21840413

[14] Vainio EJ, Korhonen K, Tuomivirta TT, Hantula J. 2010. A novel putative partitivirus of the saprotrophic fungus Heterobasidion ecrustosum infects pathogenic species of the Heterobasidion annosum complex. Fungal Biol 114:955–965. doi:10.1016/j.funbio.2010.09.006 21036340

[15] Xiao X, Cheng J, Tang J, Fu Y, Jiang D, Baker TS, Ghabrial SA, Xie J. 2014. A novel partitivirus that confers hypovirulence on plant pathogenic fungi. J Virol 88:10120–10133. doi:10.1128/JVI.01036-14 24965462 PMC4136314

[16] Chiba S, Lin Y-H, Kondo H, Kanematsu S, Suzuki N. 2013. A novel victorivirus from a phytopathogenic fungus, Rosellinia necatrix, is infectious as particles and targeted by RNA silencing. J Virol 87:6727–6738. doi:10.1128/JVI.00557-13 23552428 PMC3676089

[17] Lau SKP, Lo GCS, Chow FWN, Fan RYY, Cai JJ, Yuen K-Y, Woo PCY. 2018. Novel partitivirus enhances virulence of and causes aberrant gene expression in Talaromyces marneffei. mBio 9:e00947-18. doi:10.1128/mBio.00947-18 29895639 PMC6016240

[18] Wang Y-C, Hao X-Y, Wang L Bin Xiao Wang X-C, Yang Y-J. 2016. Diverse Colletotrichum species cause anthracnose of tea plants (Camellia sinensis (L.) O. Kuntze) in China. Sci Rep 6:35287. doi:10.1038/srep35287 27782129 PMC5080629

[19] Li Q, Zhu J, Ren N, Li D, Jin Y, Lu W, Lu Q. 2023. Characteristics and Pathogenicity of Discula theae-sinensis isolated from tea plant (Camellia sinensis) and interaction with Colletotrichum spp. Plants (Basel) 12:3427. doi:10.3390/plants12193427 37836167 PMC10574372

[20] Guo M, Zhao S, Gao Y, Shen X, Hou C. 2024. A phylogenetic and taxonomic revision of Discula theae-sinensis, the causal agents of anthracnose on Camellia sinensis. JoF 10:141. doi:10.3390/jof10020141 38392813 PMC10889989

[21] Arjona-Lopez JM, Telengech P, Jamal A, Hisano S, Kondo H, Yelin MD, Arjona-Girona I, Kanematsu S, Lopez-Herrera CJ, Suzuki N. 2018. Novel, diverse RNA viruses from Mediterranean isolates of the phytopathogenic fungus, Rosellinia necatrix: insights into evolutionary biology of fungal viruses. Environ Microbiol 20:1464–1483. doi:10.1111/1462-2920.14065 29411500

[22] Shafik K, Umer M, You H, Aboushedida H, Wang Z, Ni D, Xu W. 2021. Characterization of a novel mitovirus infecting Melanconiella theae isolated from tea plants. Front Microbiol 12:757556. doi:10.3389/fmicb.2021.757556 34867881 PMC8635788

[23] Zhou L, Li X, Kotta-Loizou I, Dong K, Li S, Ni D, Hong N, Wang G, Xu W. 2021. A mycovirus modulates the endophytic and pathogenic traits of a plant associated fungus. ISME J 15:1893–1906. doi:10.1038/s41396-021-00892-3 33531623 PMC8245556

[24] Jia H, Dong K, Zhou L, Wang G, Hong N, Jiang D, Xu W. 2017. A dsRNA virus with filamentous viral particles. Nat Commun 8:168. doi:10.1038/s41467-017-00237-9 28761042 PMC5537263

[25] Ye L, Shi X, He Y, Chen J, Xu Q, Shafik K, Fu L, Yin Y, Kotta-Loizou I, Xu W. 2023. A novel botybirnavirus with a unique satellite dsRNA causes latent infection in Didymella theifolia isolated from tea plants. Microbiol Spectr 11:e0003323. doi:10.1128/spectrum.00033-23 37962342 PMC10714997

[26] Han Z, Liu J, Kong L, He Y, Wu H, Xu W. 2023. A special satellite-like RNA of a novel hypovirus from Pestalotiopsis fici broadens the definition of fungal satellite. PLoS Pathog 19:e1010889. doi:10.1371/journal.ppat.1010889 37285391 PMC10281576

[27] Peyambari M, Habibi MK, Fotouhifar K-B, Dizadji A, Roossinck MJ. 2014. Molecular characterization of a novel putative partitivirus infecting Cytospora sacchari, a plant pathogenic fungus. Plant Pathol J 30:151–158. doi:10.5423/PPJ.OA.01.2014.0005 25288997 PMC4174853

[28] Nibert ML, Tang J, Xie J, Collier AM, Ghabrial SA, Baker TS, Tao YJ. 2013. 3D structures of fungal partitiviruses. Adv Virus Res 86:59–85. doi:10.1016/B978-0-12-394315-6.00003-9 23498903 PMC5595367

[29] Pan J, Dong L, Lin L, Ochoa WF, Sinkovits RS, Havens WM, Nibert ML, Baker TS, Ghabrial SA, Tao YJ. 2009. Atomic structure reveals the unique capsid organization of a dsRNA virus. Proc Natl Acad Sci USA 106:4225–4230. doi:10.1073/pnas.0812071106 19246376 PMC2657383

[30] Tang J, Ochoa WF, Li H, Havens WM, Nibert ML, Ghabrial SA, Baker TS. 2010. Structure of Fusarium poae virus 1 shows conserved and variable elements of partitivirus capsids and evolutionary relationships to picobirnavirus. J Struct Biol 172:363–371. doi:10.1016/j.jsb.2010.06.022 20599510 PMC3664192

[31] Ochoa WF, Havens WM, Sinkovits RS, Nibert ML, Ghabrial SA, Baker TS. 2008. Partitivirus structure reveals a 120-subunit, helix-rich capsid with distinctive surface arches formed by quasisymmetric coat-protein dimers. Structure 16:776–786. doi:10.1016/j.str.2008.02.014 18462682 PMC2556151

[32] Mahillon M, Decroës A, Caulier S, Tiendrebeogo A, Legrève A, Bragard C. 2021. Genomic and biological characterization of a novel partitivirus infecting Fusarium equiseti. Virus Res 297:198386. doi:10.1016/j.virusres.2021.198386 33716183

[33] Sasaki A, Kanematsu S, Onoue M, Oyama Y, Yoshida K. 2006. Infection of Rosellinia necatrix with purified viral particles of a member of Partitiviridae (RnPV1-W8). Arch Virol 151:697–707. doi:10.1007/s00705-005-0662-2 16307176

[34] Chiba S, Salaipeth L, Lin Y-H, Sasaki A, Kanematsu S, Suzuki N. 2009. A novel bipartite double-stranded RNA mycovirus from the white root rot fungus Rosellinia necatrix: molecular and biological characterization, taxonomic considerations, and potential for biological control. J Virol 83:12801–12812. doi:10.1128/JVI.01830-09 19828620 PMC2786845

[35] Marsh LE, Huntley CC, Pogue GP, Connell JP, Hall TC. 1991. Regulation of (+):(-)-strand asymmetry in replication of brome mosaic virus RNA. Virology (Auckl) 182:76–83. doi:10.1016/0042-6822(91)90650-Z 2024481

[36] Jurvansuu J, Kashif M, Vaario L, Vainio E, Hantula J. 2014. Partitiviruses of a fungal forest pathogen have species-specific quantities of genome segments and transcripts. Virology (Auckl) 462–463:25–33. doi:10.1016/j.virol.2014.05.021 25092458

[37] Zhu JZ, Guo J, Hu Z, Zhang XT, Li XG, Zhong J. 2021. A novel partitivirus that confer hypovirulence to the plant pathogenic fungus Colletotrichum liriopes. Front Microbiol 12:653809. doi:10.3389/fmicb.2021.653809 34248869 PMC8262616

[38] Zhong J, Chen D, Lei XH, Zhu HJ, Zhu JZ, Da Gao B. 2014. Detection and characterization of a novel gammapartitivirus in the phytopathogenic fungus Colletotrichum acutatum strain HNZJ001. Virus Res 190:104–109. doi:10.1016/j.virusres.2014.05.028 25008759

[39] Kotta-Loizou I, Coutts RHA. 2017. Studies on the virome of the entomopathogenic fungus Beauveria bassiana reveal novel dsRNA elements and mild hypervirulence. PLoS Pathog 13:e1006183. doi:10.1371/journal.ppat.1006183 28114361 PMC5293280

[40] Guo J, Zhang P, Wu N, Liu W, Liu Y, Jin H, Francis F, Wang X. 2024. Transfection of entomopathogenic Metarhizium species with a mycovirus confers hypervirulence against two lepidopteran pests. Proc Natl Acad Sci USA 121:e2320572121. doi:10.1073/pnas.2320572121 38885380 PMC11214047

[41] Wu M, Jin F, Zhang J, Yang L, Jiang D, Li G. 2012. Characterization of a novel bipartite double-stranded RNA mycovirus conferring hypovirulence in the phytopathogenic fungus Botrytis porri. J Virol 86:6605–6619. doi:10.1128/JVI.00292-12 22496220 PMC3393542

[42] Okada R, Ichinose S, Takeshita K, Urayama S-I, Fukuhara T, Komatsu K, Arie T, Ishihara A, Egusa M, Kodama M, Moriyama H. 2018. Molecular characterization of a novel mycovirus in Alternaria alternata manifesting two-sided effects: Down-regulation of host growth and up-regulation of host plant pathogenicity. Virology (Auckl) 519:23–32. doi:10.1016/j.virol.2018.03.027 29631173

[43] Fuke K, Takeshita K, Aoki N, Fukuhara T, Egusa M, Kodama M, Moriyama H. 2011. The presence of double-stranded RNAs in Alternaria alternata Japanese pear pathotype is associated with morphological changes. J Gen Plant Pathol 77:248–252. doi:10.1007/s10327-011-0315-0

[44] Cai G, Fry WE, Hillman BI. 2019. PiRV-2 stimulates sporulation in Phytophthora infestans. Virus Res 271:197674. doi:10.1016/j.virusres.2019.197674 31348964

[45] Fu M, Zhang H, Yin M, Han Z, Bai Q, Peng Y, Shafik K, Zhai L, Hong N, Xu W, Wang G, Kotta-Loizou I. 2022. A novel heptasegmented positive-sense single-stranded RNA virus from the phytopathogenic fungus Colletotrichum fructicola J Virol 96:e0031822. doi:10.1128/jvi.00318-22 35435725 PMC9093098

[46] Wang ZH, Zhao ZX, Hong N, Ni D, Cai L, Xu WX, Xiao YN. 2017. Characterization of causal agents of a novel disease inducing brown-black spots on tender tea leaves in China. Plant Dis 101:1802–1811. doi:10.1094/PDIS-04-17-0495-RE 30676920

[47] Liu H, Fu Y, Jiang D, Li G, Xie J, Peng Y, Yi X, Ghabrial SA. 2009. A novel mycovirus that is related to the human pathogen hepatitis E virus and rubi-like viruses. J Virol 83:1981–1991. doi:10.1128/JVI.01897-08 19073734 PMC2643757

[48] Kumar S, Stecher G, Tamura K. 2016. MEGA7: molecular evolutionary genetics analysis version 7.0 for bigger datasets. Mol Biol Evol 33:1870–1874. doi:10.1093/molbev/msw054 27004904 PMC8210823

[49] Xiang J, Fu M, Hong N, Zhai L, Xiao F, Wang G. 2017. Characterization of a novel botybirnavirus isolated from a phytopathogenic Alternaria fungus. Arch Virol 162:3907–3911. doi:10.1007/s00705-017-3543-6 28891001

[50] Wang L, Jiang J, Wang Y, Hong N, Zhang F, Xu W, Wang G. 2014. Hypovirulence of the phytopathogenic fungus Botryosphaeria dothidea: association with a coinfecting chrysovirus and a partitivirus. J Virol 88:7517–7527. doi:10.1128/JVI.00538-14 24760881 PMC4054428

[51] Miller LA. 2001. Microwave processing techniques for biological samples in a service laboratory, p 89–100. In Giberson RT, Demaree RS (ed), Microwave Techniques and Protocols. Humana Press, Totowa, NJ.

[52] Schneider CA, Rasband WS, Eliceiri KW. 2012. NIH Image to ImageJ: 25 years of image analysis. Nat Methods 9:671–675. doi:10.1038/nmeth.2089 22930834 PMC5554542

[53] Zhang X, Nuss DL. 2008. A host dicer is required for defective viral RNA production and recombinant virus vector RNA instability for a positive sense RNA virus. Proc Natl Acad Sci USA 105:16749–16754. doi:10.1073/pnas.0807225105 18922782 PMC2567904

[54] Fu M, Crous PW, Bai Q, Zhang PF, Xiang J, Guo YS, Zhao FF, Yang MM, Hong N, Xu WX, Wang GP. 2019. Colletotrichum species associated with anthracnose of Pyrus spp. in China. Persoonia 42:1–35. doi:10.3767/persoonia.2019.42.01 31551612 PMC6712541

[55] Kanematsu S, Arakawa M, Oikawa Y, Onoue M, Osaki H, Nakamura H, Ikeda K, Kuga-Uetake Y, Nitta H, Sasaki A, Suzaki K, Yoshida K, Matsumoto N. 2004. A reovirus causes hypovirulence of Rosellinia necatrix. Phytopathology 94:561–568. doi:10.1094/PHYTO.2004.94.6.561 18943480

[56] Yoo S-D, Cho Y-H, Sheen J. 2007. Arabidopsis mesophyll protoplasts: a versatile cell system for transient gene expression analysis. Nat Protoc 2:1565–1572. doi:10.1038/nprot.2007.199 17585298

