# Supplementary material for: A novel partitivirus confers dual contradictory effects to its host fungus: growth attenuation and virulence enhancement
Source: J Virol. 2025 Nov 24;99(12):e01219-25. doi: 10.1128/jvi.01219-25 (PMC12724381; doi:10.1128/jvi.01219-25)
Supplement: Supplemental material — Figures S1 to S3; Tables S1 to S4. [file jvi.01219-25-s0001.docx]

**Supplementary Information**
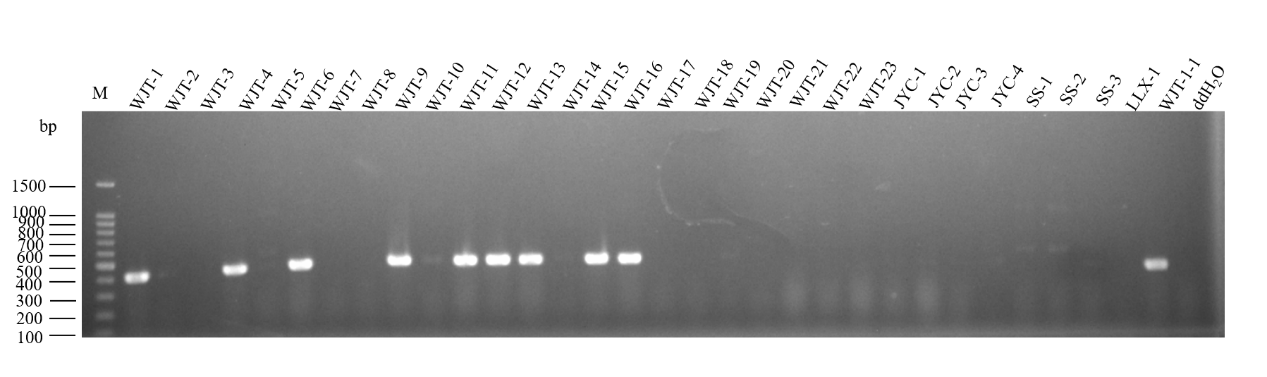


**Fig. S1 Detection of the** **ScPV1 in different strains by RT-PCR.** 31 strains were detected ScPV1with the primer pair RNA1-F2/RNA1-R2. Lane M, DNA marker; lane WJT-1-1, positive.

**
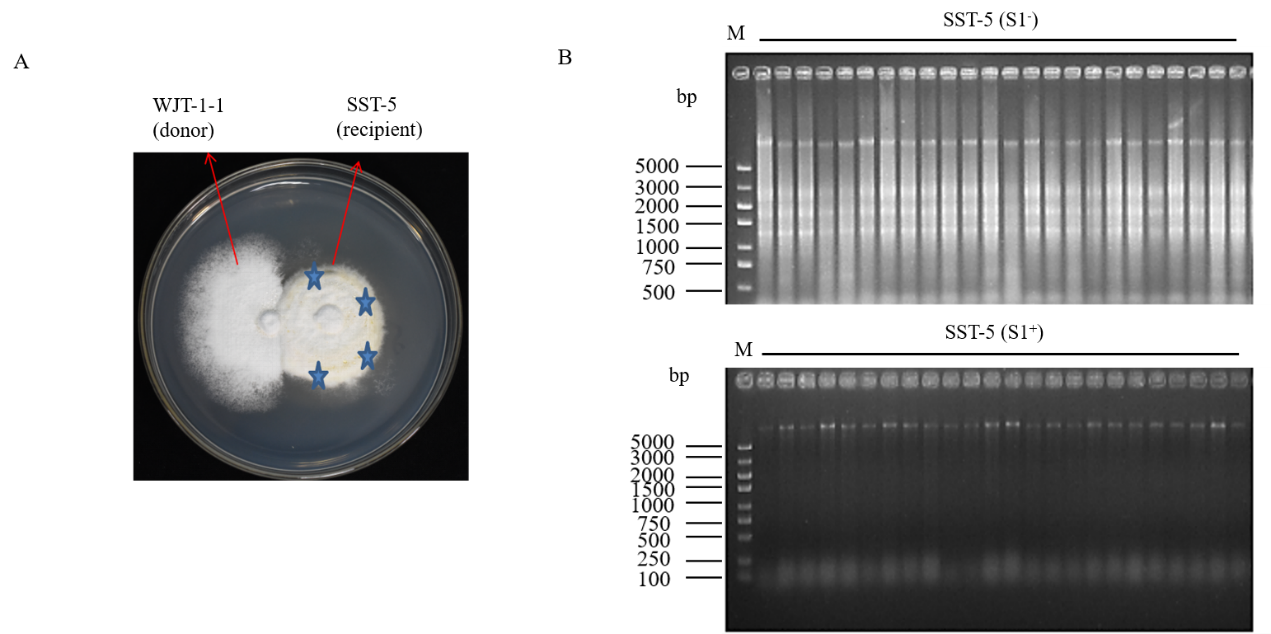
**

**Fig. S2 Horizontal transmission of ScPV1 via co-cultivation with an uninfected strain.** (A) Representative morphology of strains WJT-1-1 (donor) and SST-5 (recipient) in contact culture. (B) Agarose gel electrophoresis of nucleic acids extracted from the mycelia of 24 SST-5 subisolates derived from contact cultures, analyzed before (upper panel) and after S1 nuclease treatment (lower panel).


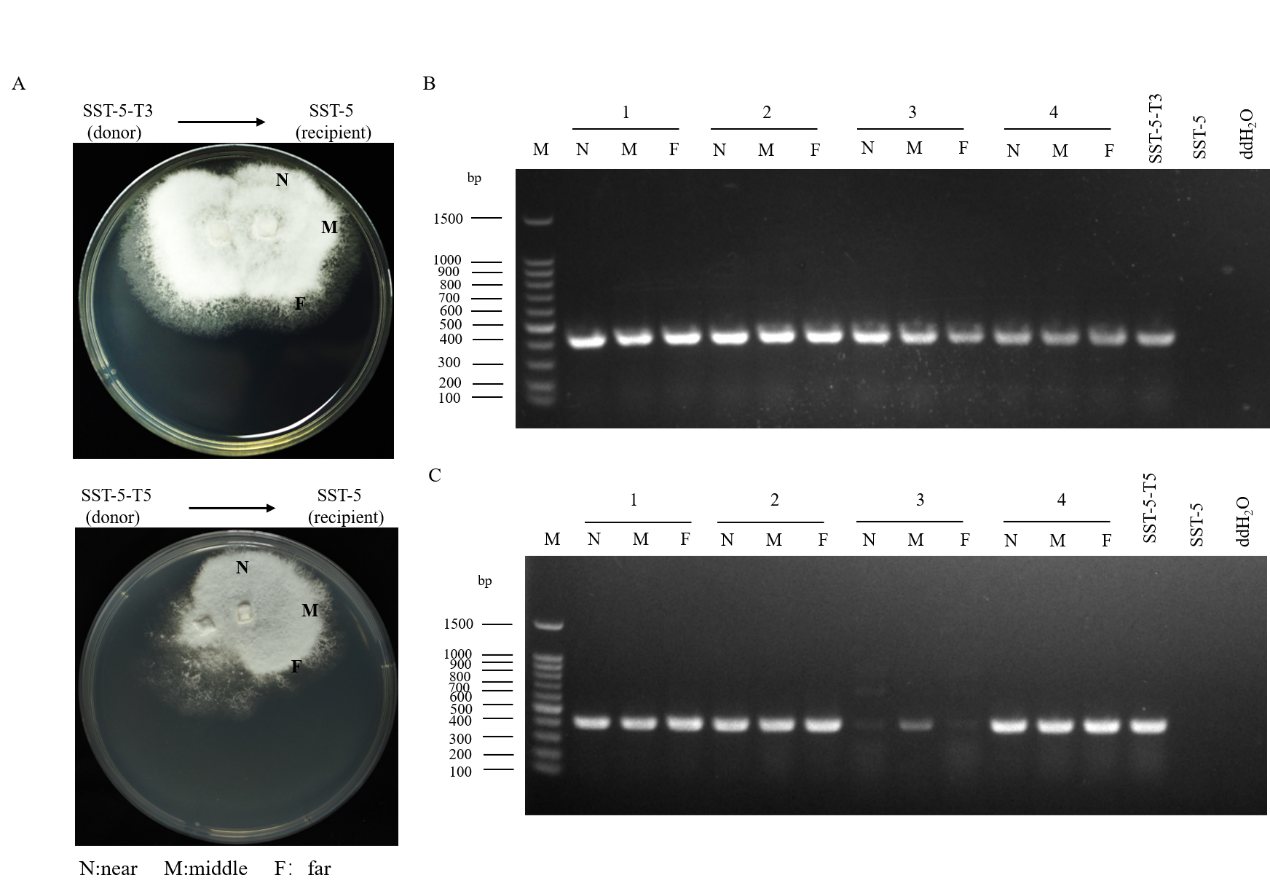
 **Fig. S3 Horizontal transmission of ScPV1 by co-cultivation with an uninfected strain.** (A) Representative morphologies of strain SST-5 (recipient) in contact culture with donor strains SST-5-T3. N, M, and F indicate sites where mycelial plugs were excised for subculture. RT-PCR detection of ScPV1 in SST-5 subisolates derived from contact cultures with SST-5-T3 (B).

**Table S1. Summary of results of a BLASTx search for full length nucleotide sequence of dsRNA1-2**

| **dsRNA No.** | **Species** | **Proteins** | **Total** **score** | **Query**  **Cover (%)** | **E** **value** | **Identity** **(%)** | **GenBank**  **accession no.** |
| --- | --- | --- | --- | --- | --- | --- | --- |
| **RNA1** | Diplodia seriata partitivirus 1 | RNA-dependent RNA polymerase | 745 | 92 | 0.0 | 62.70 | [UOK20169](https://www.ncbi.nlm.nih.gov/protein/UOK20169.1?report=genbank&log$=prottop&blast_rank=2&RID=Y46GB12S01R) |
|  | Colletotrichum eremochloae partitivirus 1 | RNA-dependent RNA polymerase | 738 | 92 | 0.0 | 62.46 | AZT88590 |
|  | Colletotrichum liriopes partitivirus 1 | RNA-dependent RNA polymerase | 758 | 93 | 0.0 | 62.00 | QZB49018 |
|  | Erysiphe necator associated partiti-like virus 1 | RNA-dependent RNA polymerase | 741 | 93 | 0.0 | 61.95 | QJW70315 |
|  | Metarhizium brunneum partitivirus 1 | RNA-dependent RNA polymerase | 734 | 92 | 0.0 | 61.71 | QHB49873 |
| **RNA2** | Colletotrichum liriopes partitivirus 1 | coat protein | 477 | 88 | 5e-160 | 52.27 | QZB49017 |
|  | Metarhizium brunneum partitivirus 1 | coat protein | 447 | 89 | 1e-148 | 48.91 | QHB49874 |
|  | Diplodia seriata partitivirus 1 | coat protein | 382 | 76 | 1e-123 | 45.21 | URN70566 |
|  | Plasmopara viticola lesion associated Partiti-like 2 | coat protein | 359 | 77 | 4e-114 | 44.97 | QGZ98413 |
|  | Erysiphe necator associated partiti-like virus 1 | coat protein | 332 | 76 | 2e-103 | 42.14 | QJW70320.1 |

**Table S2. Information of the primers used in this study**

| Primer name | Primer sequences (5' - 3') | Positions | Product length（bp） |
| --- | --- | --- | --- |
| 05RACE-3RT | CGATCGATCATGATGCAATGCNNNNNN | Random oligo for reverse transcription | / |
| 05RACE-3 | CGATCGATCATGATGCAATGC | Homologous to 05RACE-3RT | / |
| pC3-T7 loop | (P)-GGATCCCGGGAATTCGGTAATACGACTCACTATATTTTTATAGTGAGTCGTATTA-(OH) | Adaptor for RACE clonging | / |
| PC2 | CCGAATTCCCGGGATCC | Complementary to pC3-T7 loop | / |
| M13F-47 | CGCCAGGGTTTTCCCAGTCACGAC | Reverse primer for identificaion of inserts in pMD18-T plasmid | / |
| M13R-48 | AGCGGATAACAATTTCACACAGGA | Forward primer for identificaion of inserts in pMD18-T plasmid | / |
| RNA1-5'RACE-R1 | CGACTACTCTGTTGAAGGCCA | Complementary to dsRNA1 5′termini at positions 551~571 | 571 |
| RNA1-5'RACE-R2 | CCCAGACAGGACGAATTTTGG | Complementary to dsRNA1 5′termini at positions 590~610 | 610 |
| RNA1-3'RACE-F1 | AAAGAGACTGAACGAGTACAC | Homologous to dsRNA1 3′termini at positions 1464~1484 | 372 |
| RNA1-3'RACE-F2 | TCGGTCAGCTATACTCATGCA | Homologous to dsRNA1 3′termini at positions 1373~1393 | 463 |
| RNA2-5'RACE-R1 | GCAGGCGGAAGTAGAAGGTTT | Complementary to dsRNA2 5′termini at positions 675~695 | 695 |
| RNA2-5'RACE-R2 | TTGTGAGCTGAAGAACCCCAT | Complementary to dsRNA2 5′termini at positions 737~757 | 757 |
| RNA2-3'RACE-F1 | GATTCTGACCTGGCCCTCAAC | Homologous to dsRNA2 3′termini at positions 1271~1291 | 427 |
| RNA1-F1 | CCGTGCGTGGGATAGCATTGG | Homologous to dsRNA1 at positions 498~518 | 1002 |
| RNA1-R1 | GAAGGCCGGGTGGTTGTGTAC | Complementary to dsRNA1 at positions 1479~1499 |  |
| RNA2-F1 | CTTCCTGAGCATCATGGATGC | Homologous to dsRNA1 at positions 580~600 | 800 |
| RNA2-R1 | CGATCTCCGTAACGACACGG | Complementary to dsRNA1 at positions 1360~1379 |  |
| RNA1-F2 | TTACACATGCAATGAGGCGTA | Homologous to dsRNA1 at positions 554~574 | 411 |
| RNA1-R2 | CAGGTCAGAATCCAAACAACC | Complementary to dsRNA1 at positions 942~964 |  |

**Table S3. Summary of the peptide mass fingerprinting analysis of CP(P55)**

| Amino acid position | Expected Mass | Calculated Mass | ppm | Amino acid sequence | Ions score |
| --- | --- | --- | --- | --- | --- |
| 2-24 | 2320.12 | 2320.114 | 2 | ADNKTTVSGTTIGPDESTSQVGR | 78 |
| 2-24 | 2363.115 | 2363.109 | 3 | ADNKTTVSGTTIGPDESTSQVGR | 87 |
| 2-24 | 2363.122 | 2363.109 | 6 | ADNKTTVSGTTIGPDESTSQVGR | 52 |
| 2-24 | 2363.13 | 2363.109 | 9 | ADNKTTVSGTTIGPDESTSQVGR | 83 |
| 2-24 | 2363.131 | 2363.109 | 10 | ADNKTTVSGTTIGPDESTSQVGR | 77 |
| 6-24 | 1891.911 | 1891.912 | -1 | TTVSGTTIGPDESTSQVGR | 97 |
| 6-24 | 1891.915 | 1891.912 | 1 | TTVSGTTIGPDESTSQVGR | 107 |
| 6-24 | 1891.918 | 1891.912 | 3 | TTVSGTTIGPDESTSQVGR | 99 |
| 6-24 | 1891.919 | 1891.912 | 4 | TTVSGTTIGPDESTSQVGR | 96 |
| 6-24 | 1891.923 | 1891.912 | 6 | TTVSGTTIGPDESTSQVGR | 74 |
| 25-33 | 869.4367 | 869.4355 | 1 | SPAGTANPR | 58 |
| 39-64 | 2804.45 | 2804.428 | 8 | NRPSRNLKDKDAPQGAVQVQPGGQSR | 21 |
| 44-64 | 2192.129 | 2192.13 | -1 | NLKDKDAPQGAVQVQPGGQSR | 30 |
| 44-64 | 2192.13 | 2192.13 | 0 | NLKDKDAPQGAVQVQPGGQSR | 56 |
| 44-64 | 2192.132 | 2192.13 | 1 | NLKDKDAPQGAVQVQPGGQSR | 3 |
| 44-64 | 2192.134 | 2192.13 | 2 | NLKDKDAPQGAVQVQPGGQSR | 94 |
| 44-64 | 2193.117 | 2193.114 | 2 | NLKDKDAPQGAVQVQPGGQSR | 64 |
| 44-64 | 2193.119 | 2193.114 | 3 | NLKDKDAPQGAVQVQPGGQSR | 43 |
| 44-64 | 2193.124 | 2193.114 | 5 | NLKDKDAPQGAVQVQPGGQSR | 38 |
| 44-64 | 2194.115 | 2194.098 | 8 | NLKDKDAPQGAVQVQPGGQSR | 106 |
| 44-67 | 2478.302 | 2478.294 | 3 | NLKDKDAPQGAVQVQPGGQSRTGK | 12 |
| 47-64 | 1836.905 | 1836.908 | -2 | DKDAPQGAVQVQPGGQSR | 46 |
| 47-64 | 1836.905 | 1836.908 | -2 | DKDAPQGAVQVQPGGQSR | 68 |
| 47-64 | 1836.909 | 1836.908 | 1 | DKDAPQGAVQVQPGGQSR | 63 |
| 47-67 | 2123.075 | 2123.072 | 2 | DKDAPQGAVQVQPGGQSRTGK | 34 |
| 47-67 | 2123.078 | 2123.072 | 3 | DKDAPQGAVQVQPGGQSRTGK | 25 |
| 49-64 | 1593.787 | 1593.786 | 1 | DAPQGAVQVQPGGQSR | 21 |
| 49-64 | 1593.794 | 1593.786 | 5 | DAPQGAVQVQPGGQSR | 6 |
| 49-64 | 1594.778 | 1594.77 | 5 | DAPQGAVQVQPGGQSR | 37 |
| 68-90 | 2479.252 | 2479.271 | -7 | TTPVQTATIPLSGWGEIDLTSHR | 27 |
| 68-90 | 2479.274 | 2479.271 | 1 | TTPVQTATIPLSGWGEIDLTSHR | 2 |
| 68-90 | 2479.276 | 2479.271 | 2 | TTPVQTATIPLSGWGEIDLTSHR | 54 |
| 68-90 | 2479.277 | 2479.271 | 3 | TTPVQTATIPLSGWGEIDLTSHR | 70 |
| 68-90 | 2479.278 | 2479.271 | 3 | TTPVQTATIPLSGWGEIDLTSHR | 90 |
| 68-90 | 2479.28 | 2479.271 | 4 | TTPVQTATIPLSGWGEIDLTSHR | 37 |
| 68-90 | 2480.279 | 2480.255 | 10 | TTPVQTATIPLSGWGEIDLTSHR | 64 |
| 91-109 | 2198.033 | 2198.028 | 2 | NDIEPTWAPDAQPFDDLVR | 65 |
| 91-109 | 2198.034 | 2198.028 | 3 | NDIEPTWAPDAQPFDDLVR | 74 |
| 91-109 | 2198.034 | 2198.028 | 3 | NDIEPTWAPDAQPFDDLVR | 115 |
| 91-109 | 2198.038 | 2198.028 | 4 | NDIEPTWAPDAQPFDDLVR | 47 |
| 91-118 | 3232.557 | 3232.543 | 4 | NDIEPTWAPDAQPFDDLVRTTYQGLQSR | 13 |
| 110-118 | 1052.525 | 1052.525 | 0 | TTYQGLQSR | 16 |
| 110-118 | 1052.526 | 1052.525 | 1 | TTYQGLQSR | 57 |
| 110-118 | 1052.527 | 1052.525 | 2 | TTYQGLQSR | 60 |
| 110-118 | 1052.528 | 1052.525 | 2 | TTYQGLQSR | 36 |
| 110-118 | 1052.528 | 1052.525 | 2 | TTYQGLQSR | 59 |
| 110-118 | 1052.528 | 1052.525 | 3 | TTYQGLQSR | 30 |
| 110-118 | 1052.528 | 1052.525 | 3 | TTYQGLQSR | 14 |
| 110-118 | 1052.529 | 1052.525 | 3 | TTYQGLQSR | 38 |
| 110-118 | 1053.51 | 1053.509 | 1 | TTYQGLQSR | 57 |
| 110-124 | 1615.8 | 1615.795 | 3 | TTYQGLQSRFSSGGK | 30 |
| 110-124 | 1615.801 | 1615.795 | 4 | TTYQGLQSRFSSGGK | 74 |
| 143-148 | 783.4755 | 783.4755 | 0 | VLFLHR | 36 |
| 143-148 | 783.4767 | 783.4755 | 1 | VLFLHR | 36 |
| 143-148 | 783.4767 | 783.4755 | 2 | VLFLHR | 36 |
| 143-148 | 783.4769 | 783.4755 | 2 | VLFLHR | 36 |
| 143-148 | 783.4772 | 783.4755 | 2 | VLFLHR | 35 |
| 143-149 | 939.578 | 939.5766 | 1 | VLFLHRR | 12 |
| 149-159 | 1245.633 | 1245.631 | 2 | RNGNVLTTDEK | 26 |
| 149-159 | 1245.634 | 1245.631 | 2 | RNGNVLTTDEK | 38 |
| 149-159 | 1246.617 | 1246.615 | 1 | RNGNVLTTDEK | 31 |
| 149-159 | 1246.618 | 1246.615 | 2 | RNGNVLTTDEK | 39 |
| 150-159 | 1089.531 | 1089.53 | 1 | NGNVLTTDEK | 43 |
| 150-159 | 1089.533 | 1089.53 | 2 | NGNVLTTDEK | 67 |
| 150-159 | 1090.512 | 1090.514 | -2 | NGNVLTTDEK | 43 |
| 150-159 | 1090.517 | 1090.514 | 2 | NGNVLTTDEK | 65 |
| 150-159 | 1090.518 | 1090.514 | 4 | NGNVLTTDEK | 33 |
| 198-212 | 1590.825 | 1590.823 | 1 | LPRHSLGDAGNDALR | 61 |
| 198-212 | 1590.827 | 1590.823 | 2 | LPRHSLGDAGNDALR | 18 |
| 201-212 | 1224.583 | 1224.585 | -1 | HSLGDAGNDALR | 17 |
| 201-212 | 1224.585 | 1224.585 | 0 | HSLGDAGNDALR | 58 |
| 201-212 | 1224.586 | 1224.585 | 1 | HSLGDAGNDALR | 54 |
| 201-212 | 1224.587 | 1224.585 | 2 | HSLGDAGNDALR | 53 |
| 201-212 | 1224.587 | 1224.585 | 2 | HSLGDAGNDALR | 27 |
| 201-212 | 1224.587 | 1224.585 | 2 | HSLGDAGNDALR | 20 |
| 201-212 | 1224.587 | 1224.585 | 2 | HSLGDAGNDALR | 12 |
| 201-212 | 1224.588 | 1224.585 | 3 | HSLGDAGNDALR | 28 |
| 201-212 | 1224.588 | 1224.585 | 3 | HSLGDAGNDALR | 22 |
| 201-212 | 1224.589 | 1224.585 | 3 | HSLGDAGNDALR | 28 |
| 201-212 | 1224.589 | 1224.585 | 3 | HSLGDAGNDALR | 19 |
| 201-212 | 1224.589 | 1224.585 | 3 | HSLGDAGNDALR | 25 |
| 201-212 | 1224.589 | 1224.585 | 4 | HSLGDAGNDALR | 13 |
| 201-212 | 1224.589 | 1224.585 | 4 | HSLGDAGNDALR | 15 |
| 201-212 | 1224.59 | 1224.585 | 5 | HSLGDAGNDALR | 26 |
| 201-212 | 1224.591 | 1224.585 | 5 | HSLGDAGNDALR | 16 |
| 201-212 | 1224.592 | 1224.585 | 6 | HSLGDAGNDALR | 61 |
| 201-212 | 1224.593 | 1224.585 | 6 | HSLGDAGNDALR | 12 |
| 201-212 | 1225.577 | 1225.569 | 7 | HSLGDAGNDALR | 67 |
| 201-224 | 2578.16 | 2578.162 | -1 | HSLGDAGNDALRMGFFSSQDPANR | 51 |
| 201-224 | 2578.161 | 2578.162 | 0 | HSLGDAGNDALRMGFFSSQDPANR | 2 |
| 213-224 | 1355.598 | 1355.593 | 4 | MGFFSSQDPANR | 69 |
| 213-224 | 1371.593 | 1371.588 | 4 | MGFFSSQDPANR | 74 |
| 213-224 | 1371.593 | 1371.588 | 4 | MGFFSSQDPANR | 75 |
| 213-224 | 1372.581 | 1372.572 | 6 | MGFFSSQDPANR | 62 |
| 225-232 | 968.4458 | 968.4426 | 3 | ATADMWFK | 21 |
| 225-232 | 984.4404 | 984.4375 | 3 | ATADMWFK | 35 |
| 225-232 | 984.4405 | 984.4375 | 3 | ATADMWFK | 17 |
| 268-300 | 3729.743 | 3729.771 | -8 | IEPQDAAWVNAYPTNNIVGWTNDTLAPHHGSWR | 3 |
| 268-300 | 3729.776 | 3729.771 | 1 | IEPQDAAWVNAYPTNNIVGWTNDTLAPHHGSWR | 53 |
| 268-300 | 3729.779 | 3729.771 | 2 | IEPQDAAWVNAYPTNNIVGWTNDTLAPHHGSWR | 51 |
| 268-300 | 3729.784 | 3729.771 | 3 | IEPQDAAWVNAYPTNNIVGWTNDTLAPHHGSWR | 29 |
| 268-300 | 3729.784 | 3729.771 | 3 | IEPQDAAWVNAYPTNNIVGWTNDTLAPHHGSWR | 49 |
| 268-300 | 3730.787 | 3730.755 | 8 | IEPQDAAWVNAYPTNNIVGWTNDTLAPHHGSWR | 62 |
| 268-300 | 3730.787 | 3730.755 | 9 | IEPQDAAWVNAYPTNNIVGWTNDTLAPHHGSWR | 47 |
| 268-300 | 3731.771 | 3731.739 | 8 | IEPQDAAWVNAYPTNNIVGWTNDTLAPHHGSWR | 54 |
| 268-300 | 3731.771 | 3731.739 | 8 | IEPQDAAWVNAYPTNNIVGWTNDTLAPHHGSWR | 26 |
| 330-334 | 661.3194 | 661.3184 | 2 | WVSDR | 20 |
| 330-334 | 661.3196 | 661.3184 | 2 | WVSDR | 9 |
| 335-342 | 950.5425 | 950.5437 | -1 | LSTLKDFK | 22 |
| 335-342 | 950.5439 | 950.5437 | 0 | LSTLKDFK | 59 |
| 335-342 | 950.5442 | 950.5437 | 1 | LSTLKDFK | 37 |
| 335-342 | 950.545 | 950.5437 | 1 | LSTLKDFK | 31 |
| 335-342 | 950.546 | 950.5437 | 2 | LSTLKDFK | 24 |
| 335-347 | 1526.849 | 1526.846 | 2 | LSTLKDFKVFSSR | 40 |
| 335-347 | 1526.852 | 1526.846 | 4 | LSTLKDFKVFSSR | 71 |
| 335-347 | 1526.855 | 1526.846 | 6 | LSTLKDFKVFSSR | 57 |
| 348-385 | 4102.042 | 4102.013 | 7 | QLTLSTQGNPIQAYWLGTETDISNLDQRPAVASVDGNK | 28 |
| 348-385 | 4103.038 | 4102.997 | 10 | QLTLSTQGNPIQAYWLGTETDISNLDQRPAVASVDGNK | 8 |
| 386-399 | 1546.746 | 1546.741 | 3 | LNGCLDSDLALNSR | 98 |
| 386-399 | 1547.734 | 1547.725 | 6 | LNGCLDSDLALNSR | 135 |
| 400-405 | 697.3109 | 697.3105 | 1 | FAMDSK | 32 |
| 400-405 | 713.3078 | 713.3054 | 3 | FAMDSK | 21 |
| 406-416 | 1317.62 | 1317.618 | 2 | TLCPAYAFGYR | 33 |
| 406-416 | 1317.621 | 1317.618 | 2 | TLCPAYAFGYR | 43 |
| 406-416 | 1317.621 | 1317.618 | 2 | TLCPAYAFGYR | 45 |
| 406-416 | 1317.622 | 1317.618 | 3 | TLCPAYAFGYR | 45 |
| 406-416 | 1317.623 | 1317.618 | 4 | TLCPAYAFGYR | 27 |
| 420-432 | 1454.786 | 1454.784 | 1 | SRVVTEIVDGQPR | 62 |
| 420-432 | 1454.786 | 1454.784 | 1 | SRVVTEIVDGQPR | 82 |
| 420-432 | 1454.787 | 1454.784 | 2 | SRVVTEIVDGQPR | 38 |
| 420-432 | 1454.788 | 1454.784 | 2 | SRVVTEIVDGQPR | 14 |
| 420-432 | 1454.788 | 1454.784 | 3 | SRVVTEIVDGQPR | 29 |
| 422-432 | 1211.651 | 1211.651 | 0 | VVTEIVDGQPR | 39 |
| 422-432 | 1211.653 | 1211.651 | 1 | VVTEIVDGQPR | 68 |
| 422-432 | 1211.653 | 1211.651 | 2 | VVTEIVDGQPR | 59 |
| 422-432 | 1211.653 | 1211.651 | 2 | VVTEIVDGQPR | 41 |
| 422-432 | 1211.655 | 1211.651 | 3 | VVTEIVDGQPR | 46 |
| 422-432 | 1211.655 | 1211.651 | 3 | VVTEIVDGQPR | 62 |
| 422-432 | 1211.655 | 1211.651 | 3 | VVTEIVDGQPR | 56 |
| 422-432 | 1211.655 | 1211.651 | 3 | VVTEIVDGQPR | 54 |
| 422-432 | 1211.655 | 1211.651 | 3 | VVTEIVDGQPR | 46 |
| 422-432 | 1211.655 | 1211.651 | 4 | VVTEIVDGQPR | 48 |
| 422-432 | 1211.655 | 1211.651 | 4 | VVTEIVDGQPR | 59 |
| 422-432 | 1211.656 | 1211.651 | 4 | VVTEIVDGQPR | 55 |
| 422-432 | 1211.658 | 1211.651 | 6 | VVTEIVDGQPR | 45 |
| 422-436 | 1730.898 | 1730.895 | 2 | VVTEIVDGQPRYAER | 15 |
| 422-436 | 1730.899 | 1730.895 | 2 | VVTEIVDGQPRYAER | 18 |
| 437-463 | 2935.311 | 2935.324 | -4 | SNYDPWLFTNAAGTAYVDPGAAYMANR | 27 |
| 437-463 | 2935.328 | 2935.324 | 2 | SNYDPWLFTNAAGTAYVDPGAAYMANR | 24 |
| 437-463 | 2951.321 | 2951.318 | 1 | SNYDPWLFTNAAGTAYVDPGAAYMANR | 89 |
| 437-463 | 2951.327 | 2951.318 | 3 | SNYDPWLFTNAAGTAYVDPGAAYMANR | 54 |
| 437-473 | 4181.888 | 4181.872 | 4 | SNYDPWLFTNAAGTAYVDPGAAYMANRNEHFQFGSQR | 18 |
| 437-473 | 4183.861 | 4183.84 | 5 | SNYDPWLFTNAAGTAYVDPGAAYMANRNEHFQFGSQR | 6 |
| 464-473 | 1248.563 | 1248.564 | 0 | NEHFQFGSQR | 29 |
| 464-473 | 1248.564 | 1248.564 | 0 | NEHFQFGSQR | 26 |
| 464-473 | 1248.565 | 1248.564 | 1 | NEHFQFGSQR | 37 |
| 464-473 | 1248.566 | 1248.564 | 2 | NEHFQFGSQR | 48 |
| 464-473 | 1248.566 | 1248.564 | 2 | NEHFQFGSQR | 33 |
| 464-473 | 1248.566 | 1248.564 | 2 | NEHFQFGSQR | 17 |
| 464-473 | 1248.566 | 1248.564 | 2 | NEHFQFGSQR | 35 |
| 464-473 | 1248.566 | 1248.564 | 2 | NEHFQFGSQR | 32 |
| 464-473 | 1248.566 | 1248.564 | 2 | NEHFQFGSQR | 12 |
| 464-473 | 1248.567 | 1248.564 | 2 | NEHFQFGSQR | 29 |
| 464-473 | 1248.567 | 1248.564 | 2 | NEHFQFGSQR | 32 |
| 464-473 | 1248.567 | 1248.564 | 2 | NEHFQFGSQR | 35 |
| 464-473 | 1248.567 | 1248.564 | 3 | NEHFQFGSQR | 28 |
| 464-473 | 1248.567 | 1248.564 | 3 | NEHFQFGSQR | 32 |
| 464-473 | 1248.567 | 1248.564 | 3 | NEHFQFGSQR | 35 |
| 464-473 | 1248.567 | 1248.564 | 3 | NEHFQFGSQR | 36 |
| 464-473 | 1248.567 | 1248.564 | 3 | NEHFQFGSQR | 30 |
| 464-473 | 1248.568 | 1248.564 | 3 | NEHFQFGSQR | 33 |
| 464-473 | 1248.568 | 1248.564 | 3 | NEHFQFGSQR | 8 |
| 464-473 | 1248.568 | 1248.564 | 4 | NEHFQFGSQR | 30 |
| 464-473 | 1248.568 | 1248.564 | 4 | NEHFQFGSQR | 31 |
| 464-473 | 1248.568 | 1248.564 | 4 | NEHFQFGSQR | 33 |
| 464-473 | 1248.569 | 1248.564 | 4 | NEHFQFGSQR | 25 |
| 464-473 | 1248.569 | 1248.564 | 4 | NEHFQFGSQR | 33 |
| 464-473 | 1248.57 | 1248.564 | 5 | NEHFQFGSQR | 29 |
| 474-479 | 774.439 | 774.4388 | 0 | YINLPR | 32 |
| 474-479 | 774.4394 | 774.4388 | 1 | YINLPR | 27 |
| 474-479 | 774.4394 | 774.4388 | 1 | YINLPR | 27 |
| 474-479 | 774.44 | 774.4388 | 1 | YINLPR | 35 |
| 474-479 | 774.4401 | 774.4388 | 2 | YINLPR | 33 |
| 474-479 | 774.4404 | 774.4388 | 2 | YINLPR | 27 |
| 474-479 | 774.4406 | 774.4388 | 2 | YINLPR | 31 |
| 474-479 | 774.4409 | 774.4388 | 3 | YINLPR | 28 |
| 474-479 | 774.4413 | 774.4388 | 3 | YINLPR | 27 |
| 474-479 | 774.4428 | 774.4388 | 5 | YINLPR | 32 |
| 480-487 | 927.4907 | 927.4926 | -2 | FSTHALPR | 31 |
| 480-487 | 927.4927 | 927.4926 | 0 | FSTHALPR | 44 |
| 480-487 | 927.4931 | 927.4926 | 0 | FSTHALPR | 31 |
| 480-487 | 927.4931 | 927.4926 | 0 | FSTHALPR | 24 |
| 480-487 | 927.4932 | 927.4926 | 1 | FSTHALPR | 23 |
| 480-487 | 927.4933 | 927.4926 | 1 | FSTHALPR | 42 |
| 480-487 | 927.4934 | 927.4926 | 1 | FSTHALPR | 33 |
| 480-487 | 927.4934 | 927.4926 | 1 | FSTHALPR | 24 |
| 480-487 | 927.4938 | 927.4926 | 1 | FSTHALPR | 22 |
| 480-487 | 927.494 | 927.4926 | 1 | FSTHALPR | 46 |
| 480-487 | 927.4942 | 927.4926 | 2 | FSTHALPR | 26 |
| 480-487 | 927.4943 | 927.4926 | 2 | FSTHALPR | 50 |
| 480-487 | 927.4943 | 927.4926 | 2 | FSTHALPR | 34 |
| 480-487 | 927.4945 | 927.4926 | 2 | FSTHALPR | 26 |
| 480-487 | 927.4947 | 927.4926 | 2 | FSTHALPR | 30 |
| 480-487 | 927.4949 | 927.4926 | 2 | FSTHALPR | 29 |
| 480-487 | 927.4949 | 927.4926 | 2 | FSTHALPR | 38 |
| 480-487 | 927.495 | 927.4926 | 3 | FSTHALPR | 26 |
| 480-487 | 927.4951 | 927.4926 | 3 | FSTHALPR | 28 |
| 480-487 | 927.4952 | 927.4926 | 3 | FSTHALPR | 27 |
| 480-487 | 927.4953 | 927.4926 | 3 | FSTHALPR | 30 |
| 480-487 | 927.4955 | 927.4926 | 3 | FSTHALPR | 26 |
| 480-487 | 927.4959 | 927.4926 | 3 | FSTHALPR | 28 |
| 480-487 | 927.496 | 927.4926 | 4 | FSTHALPR | 29 |
| 480-487 | 927.4961 | 927.4926 | 4 | FSTHALPR | 33 |
| 480-487 | 927.4961 | 927.4926 | 4 | FSTHALPR | 27 |
| 480-487 | 927.4962 | 927.4926 | 4 | FSTHALPR | 23 |
| 480-487 | 927.4962 | 927.4926 | 4 | FSTHALPR | 23 |
| 480-487 | 927.4963 | 927.4926 | 4 | FSTHALPR | 36 |
| 480-487 | 927.4964 | 927.4926 | 4 | FSTHALPR | 28 |
| 480-487 | 927.4966 | 927.4926 | 4 | FSTHALPR | 24 |
| 480-487 | 927.497 | 927.4926 | 5 | FSTHALPR | 26 |
| 480-501 | 2283.224 | 2283.222 | 1 | FSTHALPRSAALDAAIVLSDTK | 1 |
| 488-501 | 1373.743 | 1373.74 | 2 | SAALDAAIVLSDTK | 59 |
| 488-501 | 1373.743 | 1373.74 | 2 | SAALDAAIVLSDTK | 97 |
| 488-501 | 1373.743 | 1373.74 | 2 | SAALDAAIVLSDTK | 91 |
| 488-501 | 1373.744 | 1373.74 | 3 | SAALDAAIVLSDTK | 87 |
| 488-501 | 1373.744 | 1373.74 | 3 | SAALDAAIVLSDTK | 91 |
| 488-501 | 1373.745 | 1373.74 | 4 | SAALDAAIVLSDTK | 72 |
| 488-501 | 1373.745 | 1373.74 | 4 | SAALDAAIVLSDTK | 90 |

Note: Ions score is −10 log(*P*), where *P* is the probability that the observed match is a random event. Protein scores are derived from ions scores as a non-probabilistic basis for ranking protein families. Individual ions scores > 0 indicate identity or extensive homology (p<0.05). The overall protein score is 6586. ppm means peptide mass tolerance.

**Table S4. Summary of the** **peptide mass fingerprinting analysis of RdRp (P100)**

| Amino acid position | Expected Mass | Calculated Mass | ppm | Amino acid sequence | Ions score |
| --- | --- | --- | --- | --- | --- |
| 64-71 | 961.4615 | 961.4617 | 0 | YDNNLPAR | 41 |
| 84-93 | 1213.643 | 1213.6415 | 1 | TLDELRPSQR | 8 |
| 84-93 | 1213.6439 | 1213.6415 | 2 | TLDELRPSQR | 24 |
| 111-126 | 1782.859 | 1782.8577 | 1 | TTSPGFPWVTQGYQSK | 20 |
| 173-189 | 2011.0968 | 2011.0891 | 4 | IRPVWGYPLDVIVEEAR | 28 |
| 190-201 | 1580.7727 | 1580.7697 | 2 | FFFPLMEHLEQK | 4 |
| 202-217 | 1923.7637 | 1923.7615 | 1 | NNEDDTFYGLGMETMR | 16 |

Note: Ions score is −10 log(*P*), where *P* is the probability that the observed match is a random event. Protein scores are derived from ions scores as a non-probabilistic basis for ranking protein families. Individual ions scores > 0 indicate identity or extensive homology (p<0.05). The overall protein score is 76. ppm means peptide mass tolerance.
